# Fas-associated factor 1 mediates NADPH oxidase-induced reactive oxygen species production and proinflammatory responses in macrophages against Listeria infection

**DOI:** 10.1371/journal.ppat.1008004

**Published:** 2019-08-14

**Authors:** Tae-Hwan Kim, Hyun-Cheol Lee, Jae-Hoon Kim, C. Y. Hewawaduge, Kiramage Chathuranga, W. A. Gayan Chathuranga, Pathum Ekanayaka, H. M. S. M. Wijerathne, Chul-Joong Kim, Eunhee Kim, Jong-Soo Lee

**Affiliations:** 1 College of Veterinary Medicine, Chungnam National University, Daejeon, Republic of Korea; 2 Laboratory Animal Resource Center, Korea Research Institute of Bioscience and Biotechnology, University of Science and Technology (UST), Daejeon, Republic of Korea; 3 College of Biological Sciences and Biotechnology, Chungnam National University, Daejeon, Republic of Korea; University of Pennsylvania, UNITED STATES

## Abstract

Fas-associated factor 1 is a death-promoting protein that induces apoptosis by interacting with the Fas receptor. Until now, FAF1 was reported to interact potentially with diverse proteins and to function as a negative and/or positive regulator of several cellular possesses. However, the role of FAF1 in defense against bacterial infection remains unclear. Here, we show that FAF1 plays a pivotal role in activating NADPH oxidase in macrophages during *Listeria monocytogenes* infection. Upon infection by *L*. *monocytogenes*, FAF1 interacts with p67phox (an activator of the NADPH oxidase complex), thereby facilitating its stabilization and increasing the activity of NADPH oxidase. Consequently, knockdown or ectopic expression of FAF1 had a marked effect on production of ROS, proinflammatory cytokines, and antibacterial activity, in macrophages upon stimulation of TLR2 or after infection with *L*. *monocytogenes*. Consistent with this, FAF1^gt/gt^ mice, which are knocked down in FAF1, showed weaker inflammatory responses than wild-type mice; these weaker responses led to increased replication of *L*. *monocytogenes*. Collectively, these findings suggest that FAF1 positively regulates NADPH oxidase-mediated ROS production and antibacterial defenses.

## Author summary

Phagocytic NADPH oxidase plays a pivotal role in generating reactive oxygen species (ROS) and in defense against bacterial infections such as *L*. *monocytogenes*. ROS eliminate phagocytosed bacteria directly and are implicated in transduction of signals that mediate inflammatory responses. Here, we show that the apoptotic protein FAF1 regulates ROS production in macrophages by regulating phagocytic NADPH oxidase activity upon infection by *L*. *monocytogenes*. FAF1 interacts directly with and stabilizes p67phox, a regulatory protein of the phagocytic NADPH oxidase complex, to induce ROS production during *L*. *monocytogenes* infection. Production of ROS leads to release of proinflammatory cytokines, chemokines and, ultimately, to bacterial clearance. Interestingly, FAF1^gt/gt^ mice deficient in FAF1 expression exhibit weakened inflammatory responses and are thus more vulnerable to bacterial infection than FAF1^+/+^ mice. This study reveals that FAF1 is a crucial regulator that induces inflammatory responses to bacterial infection via ROS production.

## Introduction

Innate immune cells are the first barrier encountered by invading microbial pathogens. Among these cells, phagocytes such as macrophages and neutrophils play key roles in host protection against bacterial infection. Upon recognition and phagocytosis of bacteria, phagocytes produce reactive oxygen species (ROS) that kill and inactivate bacteria directly. This mechanism is known as the respiratory burst. NADPH oxidase, one of several ROS sources, is critical for this process [1, 2]. The redox center of phagocytic NADPH oxidase is a heterodimer comprising transmembrane-associated protein subunits p22phox and gp91phox (Nox2). This heterodimer, also known as flavocytochrome b588, forms a phagocytic NADPH oxidase complex together with the cytosolic regulatory subunits p67phox, p47phox, p40phox, and the small GTPase Rac [1, 3, 4]. The ROS generated by the NADPH oxidase complex are not only toxic to the cell but also participate in host defense responses such as NF-kB activation and release of proinflammatory cytokines [5]. A life-threatening genetic disorder called chronic granulomatous disease (CGD), in which the phagocytic NADPH oxidase is dysfunctional, leads to life-threatening bacterial and fungal infections. CGD is caused by mutations in any one of the genes that encode subunits of the phagocytic NADPH oxidase complex [6–8].

Upon phagocytosis of bacteria, toll-like receptors (TLRs), which are transmembrane receptors that play a critical role in innate immune recognition of pathogens, act as a first line of host defense [9, 10]. The TLR family in humans and mice includes more than ten different members, all of which have been studied extensively with respect to infection. Phagocytes express all TLR members, stimulation of which induces diverse biological processes, including inflammation, antigen presentation, and direct bactericidal effects [10, 11]. The interplay between these TLR recognition and activation of NADPH oxidase during phagocytosis of bacteria is well characterized. Especially, interaction between Nox2 and TLR2 is required for ROS production and inflammatory responses during mycobacteria infection [12–14]. Moreover, TLR2 mediates expression of Nox2 in microglia during peripheral nerve injury [15]. Nox4 is also required for TLR4-mediated ROS production in response to lipopolysaccharide (LPS) [16]. Except for bacterial infection, Nox4 is necessary for generation of macrophage migration inhibitory factor during parasite infection [17]. Recent studies show that the TLR4-Nox1 redox signaling axis plays a role in metastasis of colon cancer and lung cancer cells [18, 19].

Fas-associated factor 1 (FAF1) was identified initially in a yeast two-hybrid assay using the cytoplasmic domain of Fas protein as bait [20]. FAF1, which contains a Fas-interacting domain (FID), a death effector domain-interacting domain (DEDID), and a C-terminal domain [21] potentiates Fas-mediated apoptosis as a member of the death-inducing signaling complex [22, 23]. FAF1 interacts with different molecules and is involved in a variety of biological processes; it plays a role in regulating cell death and/or tumor progression, ubiquitination-mediated proteosomal degradation, chaperones, NF-kB signaling, and interferon signaling [24–29].

To better understand the biological role of FAF1, we examined the relationship between NADPH oxidase and FAF1 in host defense against bacterial infection. We show that FAF1 is a crucial positive regulator of the phagocytic NADPH oxidase complex, which promotes ROS production by macrophages in response to *L*. *monocytogenes* infection. FAF1 controls phagocytic NADPH oxidase-mediated inflammatory responses upon *L*. *monocytogenes* infection by interacting with p67phox, thereby inhibiting bacterial growth.

## Results

### Knockdown of FAF1 weakens antibacterial immune responses both *in vivo* and *in vitro*

**Fig 1:**
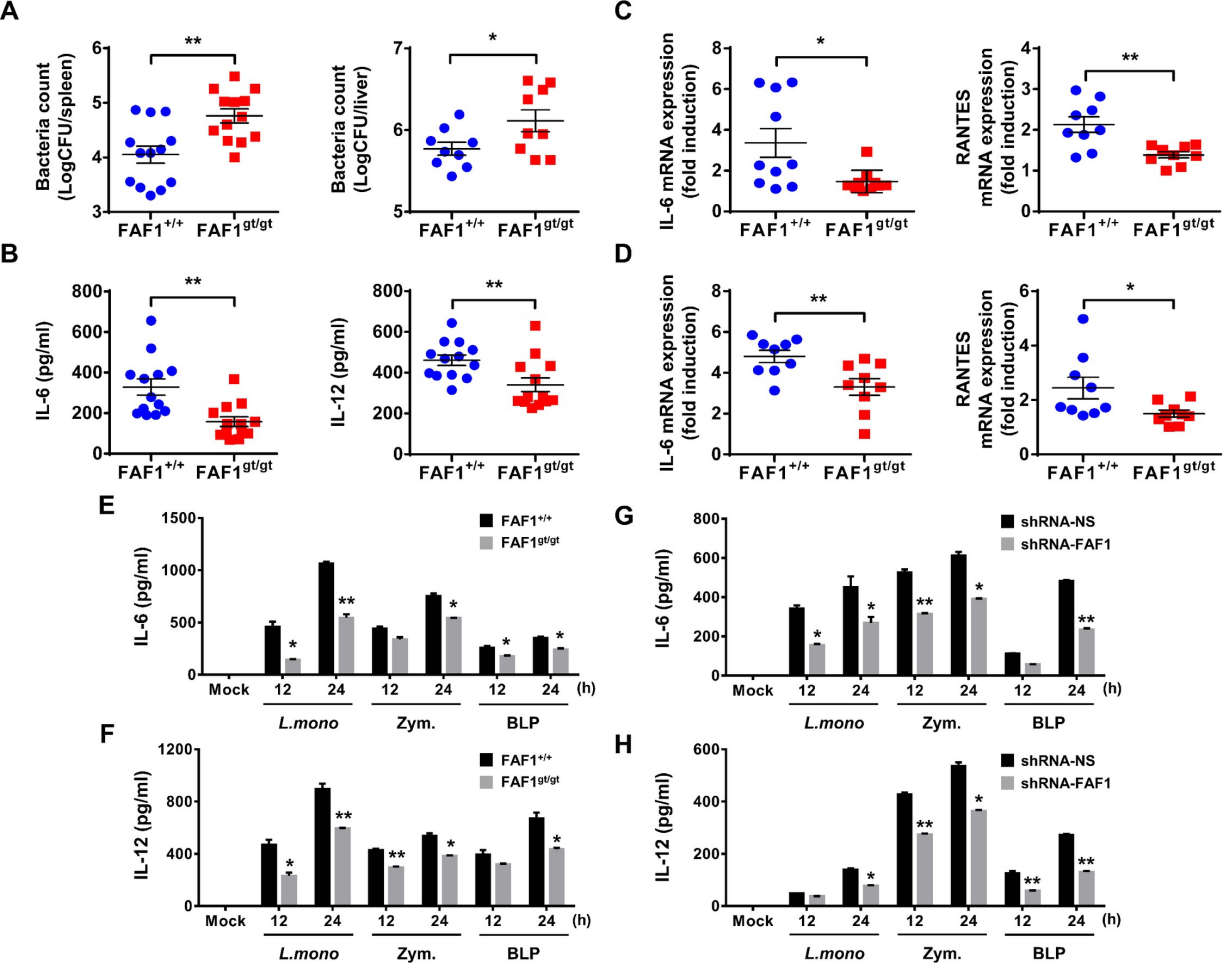
FAF1 functions as a positive regulator of antibacterial immunity *in vivo* and *in vitro*. (A-D) FAF1^+/+^ and FAF1^gt/gt^ mice were infected intraperitoneally with *L*. *monocytogenes* (5.0 × 10^5^ CFU/mouse). The bacterial load in spleens and livers from infected mice (A), serum cytokine levels (B), mRNA levels for cytokines in spleens (C) and livers (D) were measured at 24hr post-infection (n = 9–13 per group). * P < 0.05, ** P <0.01 (Mann-Whitney test). Data are the mean ± SEM of individual values pooled from two independent experiments. (E and F) FAF1^+/+^ and FAF1^gt/gt^ BMDMs were infected with *L*. *monocytogenes* (MOI = 1) for 2hrs or treated with BLP (100ng/ml) or zymosan (100μg/ml) for the indicated times and the supernatants were analyzed for levels of IL-6 (E), IL-12 (F). (G and H) Control Raw264.7 cells (shRNA-NS) and FAF1 knockdown Raw264.7 cells (shRNA-FAF1) were infected with *L*. *monocytogenes* (MOI = 1) for 2hrs or treated with BLP (100ng/ml) or zymosan (100μg/ml) for the indicated times and the supernatants were analyzed for levels of IL-6 (G), IL-12 (H). Data are representative of three (E-H) independent experiments. Error bars, mean ± SD. * P < 0.05, ** P < 0.01 (Student`s *t*-test), compared to FAF1^+/+^.

Based on a previous study of the role of FAF1 in antiviral responses against infection by RNA virus [27], we asked whether FAF1 is also involved in responses to bacterial infection. To examine *in vivo* host responses to infection by *L*. *monocytogenes*, FAF1^+/+^ and FAF1^gt/gt^ mice were infected intraperitoneally with *L*. *monocytogenes* (5 × 10^5^ CFU per mouse) and serum cytokine levels, bacterial load, and proinflammatory gene expression in the spleen and liver were measured at 24 h post-infection (hpi). The bacterial load in the spleen and liver of FAF1^gt/gt^ mice was approximately 10-fold and 3-fold higher, respectively, than that in FAF1^+/+^ mice (Fig 1, panel A). Serum cytokine levels were also reduced markedly in FAF1^gt/gt^ mice (Fig 1, panel B). Expression of proinflammatory genes in the spleen and liver of FAF1^gt/gt^ mice was also lower than that in FAF1^+/+^ mice (Fig 1, panels C-D). To determine whether these effects were mediated by peritoneal macrophages in response to *L*. *monocytogenes* infection, FAF1^+/+^ and FAF1^gt/gt^ mice were infected with *L*. *monocytogenes* intraperitoneally and peritoneal macrophages (PMs) were isolated at 24 hpi. Expression of proinflammatory cytokines and chemokines by these cells was measured by quantitative real-time PCR. Expression of mRNA encoding IL-6, CXCL10, and RANTES was significantly lower in PMs from FAF1^gt/gt^ mice than in those from FAF1^+/+^ mice (S1 Fig). These data suggest that FAF1 plays an important role in host defense against *L*. *monocytogenes* infection *in vivo*. Next, to determine whether FAF1 is involved in inflammatory responses, we examined induction of FAF1 in macrophages exposed to *L*. *monocytogenes*. Importantly, mRNA expression of FAF1 was induced with 2–3 folds at 15 or 60 m post-infection (mpi) with *L*. *monocytogenes* in Raw264.7 cells, suggesting that FAF1 responds to bacterial infection at early time (S2 Fig, panel A). Also, FAF1 responded to high MOI of bacterial infection in mouse bone marrow-derived macrophages (BMDMs) and Raw264.7 cells (S2 Fig, panels B-C). At early time points after *L*. *monocytogenes* infection, host defense is regulated by secretion of several cytokines and chemokines, including IL-6, IL-12, and RANTES [30, 31]. Therefore, we examined the effect of FAF1 on proinflammatory cytokine secretion by BMDMs and resident PMs against *L*. *monocytogenes* infection or TLR2 ligands. First, expression of FAF1 was confirmed by immunoblotting of extracts from BMDMs or PMs isolated from FAF1^+/+^ and FAF1^gt/gt^ mice (S3 Fig, panels A-B). Next, cells were infected with *L*. *monocytogenes* or treated with zymosan or bacterial lipoprotein (BLP). The supernatants were harvested at 12 or 24 hpi to measure IL-6 and IL-12 by ELISA. As results, FAF1 knockdown reduced cytokine production by both BMDMs (Fig 1, panels E-F) and PMs (S3 Fig, panels C-D). Next, we generated control and FAF1-knockdown murine macrophage cells (Raw264.7) using lentiviruses harboring non-specific or FAF1-specific small hairpin RNA (shRNA). Expression of FAF1 was then determined by immunoblot analysis (S4 Fig, panel A). Next, cells were infected with *L*. *monocytogenes* or treated with zymosan or BLP and supernatants harvested at 12 or 24 hpi to measure cytokine secretion. Consistent with the results obtained from primary cells, shRNA-mediated knockdown of FAF1 led to a marked reduction in secretion of proinflammatory cytokines IL-6 and IL-12 upon stimulation via TLR2 (Fig 1, panels G-H). Additionally, production of chemokines regulated upon activation (i.e., normal T cell expressed and secreted (RANTES) and monocyte chemoattractant protein (MCP)-1) was lower in FAF1-knockdown cells than in control cells (S4 Fig, panels B-C). Collectively, these results suggest that FAF1 expression has a marked effect on host defense responses against *L*. *monocytogenes*.

### Knockdown of FAF1 attenuates proinflammatory responses during *L*. *monocytogenes* infection to macrophages

**Fig 2:**
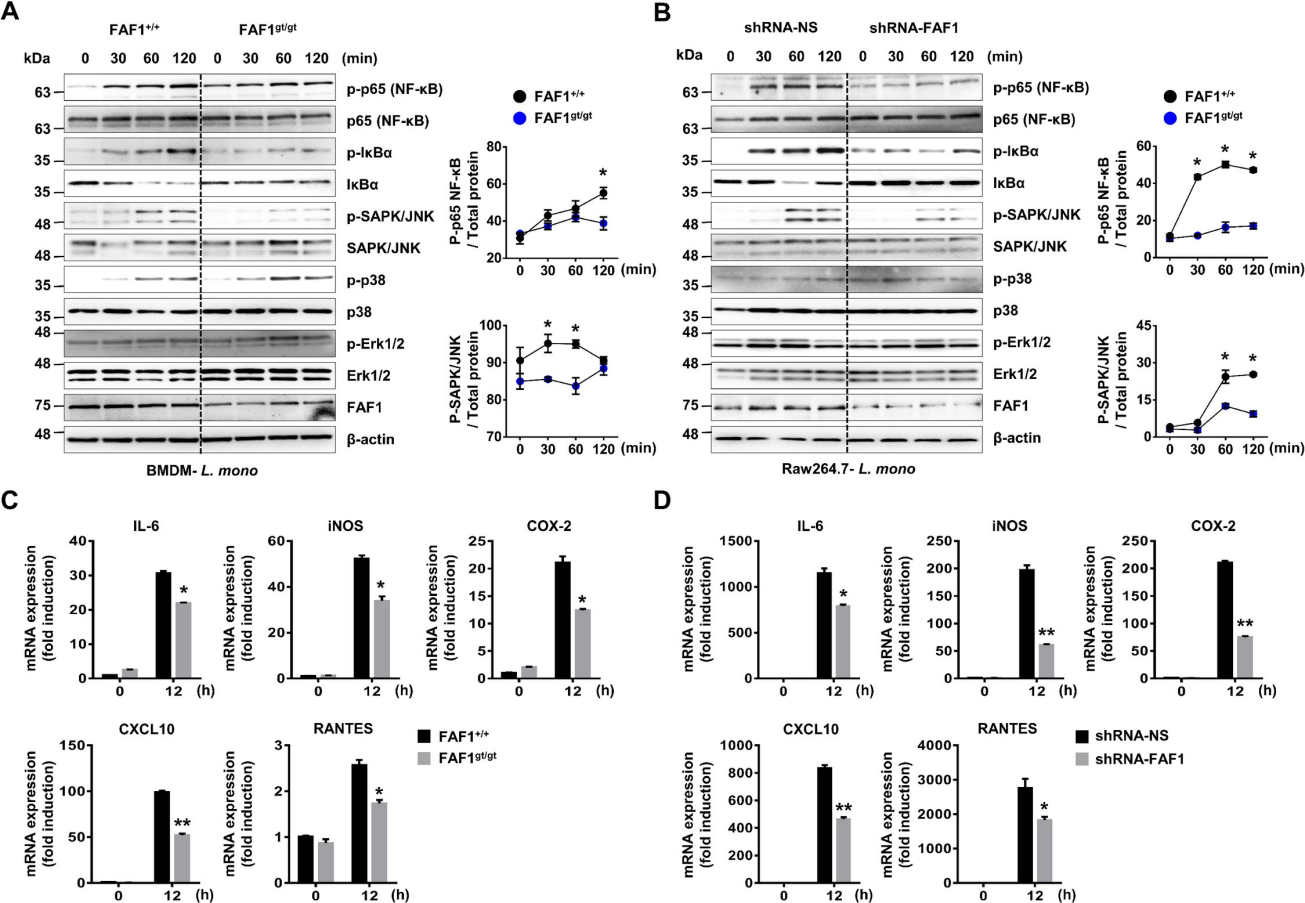
Knockdown of FAF1 reduces proinflammatory responses upon infection with *L*.*monocytogenes* in macrophages. (A) FAF1^+/+^ and FAF1^gt/gt^ BMDMs were infected with *L*. *monocytogenes* (MOI = 1) for the indicated times, and subjected to immunoblotting with phosphorylated and total forms of p65, IκBα, JNK, p38, p42/p44 MAPK, FAF1 and β-actin (left). Band intensity of each blot was shown in graphs (Right). (B) Control Raw264.7 cells (shRNA-NS) and FAF1 knockdown Raw264.7 cells (shRNA-FAF1) were infected with *L*. *monocytogenes* (MOI = 1) for the indicated times, and then subjected to immunoblotting with the phosphorylated and total forms of p65, IκBα, JNK, p38, p42/p44 MAPK, FAF1 or β-actin (left). Band intensity of each blot was shown in graphs (Right). (C) FAF1^+/+^ and FAF1^gt/gt^ BMDMs were infected with *L*. *monocytogenes* (MOI = 3) for 2hrs. At 12 hpi, cells were harvested and analyzed by quantitative real-time PCR analysis for IL-6, iNOS, COX-2, CXCL10, and RANTES genes. (D) Control Raw264.7 cells (shRNA-NS) and FAF1 knockdown Raw264.7 cells (shRNA-FAF1) were infected with *L*. *monocytogenes* (MOI = 1) for 2hrs. At 12 hpi, cells were harvested and analyzed by quantitative real-time PCR analysis for IL-6, iNOS, COX-2, CXCL10, and RANTES genes. Data are representative of two (A, B) or three (C, D) independent experiments. Error bars, mean ± SD. * P < 0.05, ** P < 0.01 (Student`s *t*-test), compared to control (FAF1^+/+^ or shRNA-NS).

To determine whether FAF1 activates proinflammatory signaling pathways, we performed immunoblot analysis to examine expression of activated forms of molecules related to the NF-κB and MAPK signaling pathways in BMDMs isolated from FAF1^+/+^ and FAF1^gt/gt^ mice infected with *L*. *monocytogenes*. The result showed that knockdown of FAF1 leads to a marked reduction in phosphorylation of p65 (NF-κB), IκBα, and SAPK/JNK, but had no effect on activation of p38 MAPK or Erk1/2 (Fig 2, panel A). In agreement with this, knockdown of FAF1 in Raw264.7 cells suppressed activation of p65 (NF-κB), IκBα, and SAPK/JNK, but not p38 or Erk1/2 MAPK (Fig 2, panel B). Furthermore, to measure expression of proinflammatory genes, BMDMs and resident PMs isolated from FAF1^+/+^ and FAF1^gt/gt^ mice were infected with *L*. *monocytogenes* and subjected to real-time PCR at 12 hpi to detect *Il6*, *Nos2* (iNOS), *Ptgs2* (COX-2), *Cxcl10*, and *RANTES*. The expression of these genes in FAF1-knockdown BMDMs cells were lower than those in wild-type cells (Fig 2, panel C) or resident PMs (S5 Fig). Consistent with this, similar results were obtained from FAF1-knockdown Raw264.7 cells (Fig 2, panel D). Taken together, these data suggest that FAF1 plays a role in inflammatory responses against *L*. *monocytogenes* infection.

### Interaction between FAF1 and p67phox leads to enhanced ROS production and bacterial clearance upon infection by *L*. *monocytogenes* in macrophages

**Fig 3:**
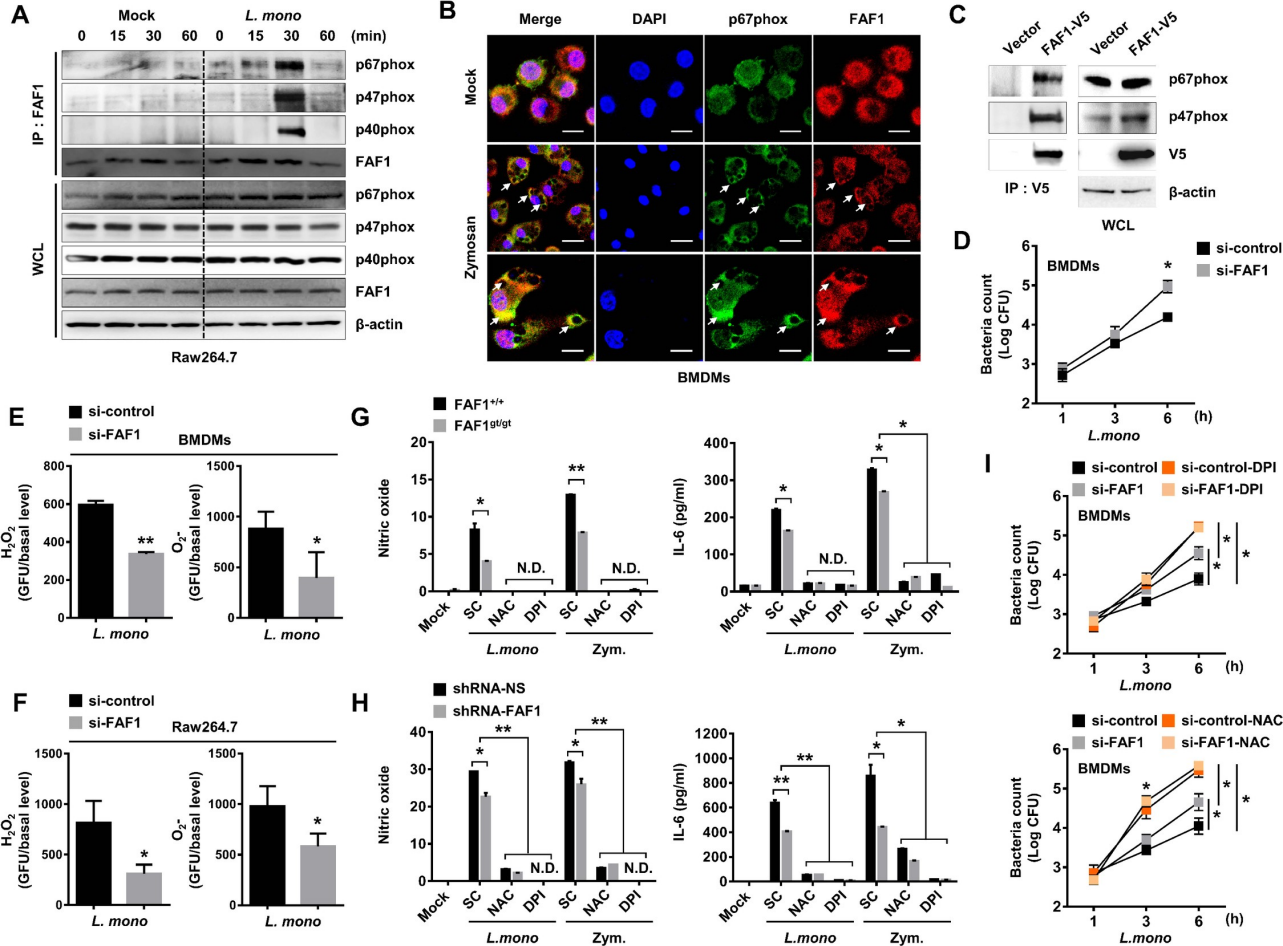
FAF1 interacts with p67phox, leading to inflammatory responses following *L*. *monocytogenes* infection. (A) Mock- or *L*. *monocytogenes-*infected Raw264.7 cells were harvested at indicated times and cell lysates were used for immunoprecipitation (IP) with an anti-FAF1 antibody, followed by immunoblotting with anti-p67phox, p47phox, p40phox, and FAF1 antibodies. (B) BMDMs were treated with mock or zymosan (100μg/ml) for 30min, followed by confocal microscopy with an anti-p67phox antibody and an anti-FAF1 antibody. Scale bars represent 10μm. (C) Raw264.7 cells containing empty vector or V5-tagged FAF1 were used for IP with an anti-V5 antibody, followed by immunoblotting with anti-p67phox, p47phox antibodies. (D) At 36hr post-transfection with siRNA-control or siRNA-FAF1, BMDMs were infected with *L*. *monocytogenes* (MOI = 0.1) for 1hr. Bacteria-containing medium was replaced with fresh medium containing gentamycin. Then, cells were washed with PBS and lysed by Triton X-100 at the indicated time, followed by CFU titration for counting intracellular bacteria. (E-F) At 36hr post-transfection with siRNA-control or siRNA-FAF1, BMDMs (E) or Raw264.7 cells (F) were infected with *L*. *monocytogenes*, then used for measuring H_2_O_2_ or O_2_^-^ production. (G) FAF1^+/+^ and FAF1^gt/gt^ BMDMs were infected with *L*. *monocytogenes* (MOI = 1) for 2hrs or stimulated with zymosan w/ or w/o NAC or DPI (20μM). At 18hr, the supernatants were analyzed for NO production or IL-6 secretion. (H) Control Raw264.7 cells (shRNA-NS) and FAF1 knockdown Raw264.7 cells (shRNA-FAF1) were infected with *L*. *monocytogenes* (MOI = 1) for 2hrs or stimulated with zymosan w/ or w/o NAC or DPI (20μM). At 18hr, the supernatants were analyzed for NO production or IL-6 secretion. (I) At 36hr post-transfection with siRNA-control or siRNA-FAF1, BMDMs were infected with *L*. *monocytogenes* (MOI = 0.1) for 1hr. Bacteria-containing medium was replaced with fresh medium containing gentamycin w/ or w/o DPI or NAC. Then, cells were washed with PBS and lysed by Triton X-100 at the indicated time, followed by CFU titration for counting intracellular bacteria. Data are representative of two (A, C, D, G, H, I) or three (B, E, F) independent experiments. Error bars, mean ± SD. * P < 0.05, ** P < 0.01 (Student`s *t*-test), compared to FAF1^+/+^ or control (si-control or shRNA-NS). N.S., no significant; N.D., no detection.

To identify the target protein of FAF1, large-scale cultured HEK293 cells were used for immunoprecipitation with an anti-FAF1 antibody, followed by mass spectrometry analysis. The result identified NADPH oxidase activator 1 (NoxA1) (S6 Fig). NoxA1 regulates activation of Nox1, which can generate ROS and is expressed at high levels in colon cancer cells [3]. As a homolog of NoxA1, p67phox acts mainly as an activator of Nox2 in phagocytes. Moreover, there is a high degree of domain homology between NoxA1 and p67phox, although the proteins show only 28% amino acid identity [3, 32, 33]. Based on these reports, we asked whether FAF1 interacts with p67phox to regulate ROS production in phagocytes. Mock- or *L*. *monocytogenes*-infected Raw264.7 cells were harvested at various time points and cell lysates were immunoprecipitated with an anti-FAF1 antibody, followed by immunoblotting with antibodies against components of the phagocytic NADPH oxidase complex (Fig 3, panel A). The result indicated that FAF1 transiently interacts with p67phox, p47phox, and p40phox at 30 mpi with *L*. *monocytogenes* (Fig 3, panel A). To test whether FAF1 expression affects those interaction, efficiency of FAF1-specific siRNA was priorly determined in Raw264.7 cells or BMDMs for further experiments (S7 Fig, panels A-B). FAF1 knockdown-BMDMs showed a weak interaction between both molecules as well as lower expression of p67phox compared with control BMDMs upon *L*. *monocytogenes* infection (S8 Fig, panel A). Similar result was obtained following siRNA-mediated knockdown of FAF1 in Raw264.7 (S8 Fig, panel B). Moreover, confocal microscopy analysis exhibited that FAF1 is translocated to phagosomal membranes upon zymosan treatment in BMDMs where it co-localizes with p67phox (Fig 3, panel B). Additionally, immunoprecipitation with an anti-V5 antibody using FAF1-overexpressing Raw264.7 cells showed strong interaction between ectopic FAF1 and endogenous p67phox without stimulation, suggesting that FAF1 might present high affinity to p67phox (Fig 3, panel C). Next, we performed CFU assay to examine the growth rate of intracellular *L*. *monocytogenes* in BMDMs following siRNA-mediated knockdown of FAF1. Knockdown of FAF1 showed a significant increase of bacterial growth compared to control BMDMs (Fig 3, panel D). This result encouraged us to verify that reduced ROS production and inflammation in FAF1-knockdown cells might result in a more favorable environment for *L*. *monocytogenes* replication. To investigate whether FAF1 affects ROS production upon *L*. *monocytogenes* infection, we used fluorescence absorbance to measure ROS production following siRNA-mediated knockdown of FAF1 in BMDMs. As expected, H_2_O_2_ and O_2_-produced by FAF1-knockdown BMDMs were significantly lower than those produced by control BMDMs in response to *L*. *monocytogenes* infection (Fig 3, panel E). Similar results were obtained from Raw264.7 cells following siRNA-mediated knockdown of FAF1 (Fig 3, panel F). Moreover, knockdown of FAF1 exhibited lower ROS production upon stimulation with zymosan in BMDMs, Raw264.7, and PMs compared to control cells. (S9 Fig, panels A-C). ROS acts as a signal transduction mediator in response to diverse stimuli [5]. To determine further whether proinflammatory cytokine production in response to *L*. *monocytogenes* infection or TLR2 signaling correlated directly with ROS generation in the presence or absence of FAF1, we measured NO and IL-6 levels in supernatants from BMDMs (Fig 3, panel G) and Raw264.7 cells (Fig 3, panel H) stimulated with *L*. *monocytogenes* or zymosan in the presence/absence of ROS inhibitors [N-acetyl-L-cysteine and diphenyleneiodonium]. As expected, treatment with ROS inhibitors reduced NO production toward nearly basal level despite of stimulation with *L*. *monocytogenes* or zymosan. Secretion of IL-6 by these cells also fell markedly, regardless of FAF1 expression. In other words, impaired ROS production resulted in no significant difference in cytokine secretion by wild-type and FAF1-knockdown macrophages. Knockdown of FAF1 attenuates IκBα degradation and NF-κB activation, but not phosphorylation of IKKs, suggesting that FAF1 indirectly regulates the inflammatory responses via ROS production on TLR2 signaling (S10 Fig). It was also supported by evidences that FAF1 augments inflammatory responses depending on NADPH oxidase complex (S11 Fig, panels A-B). Likewise, knockdown of FAF1 led to increased bacterial growth than control in BMDMs. However, there was no differences under DPI or NAC treatment, which suggests that FAF1 inhibits bacterial growth by mediating ROS production (Fig 3, panel I). Taken together, these findings demonstrate that FAF1 enhances inflammatory responses and intracellular bacterial clearance via ROS generation by interacting with p67phox upon infection by *L*. *monocytogenes*.

### FAF1 directly interacts with p67phox via amino acids 330–489

**Fig 4:**
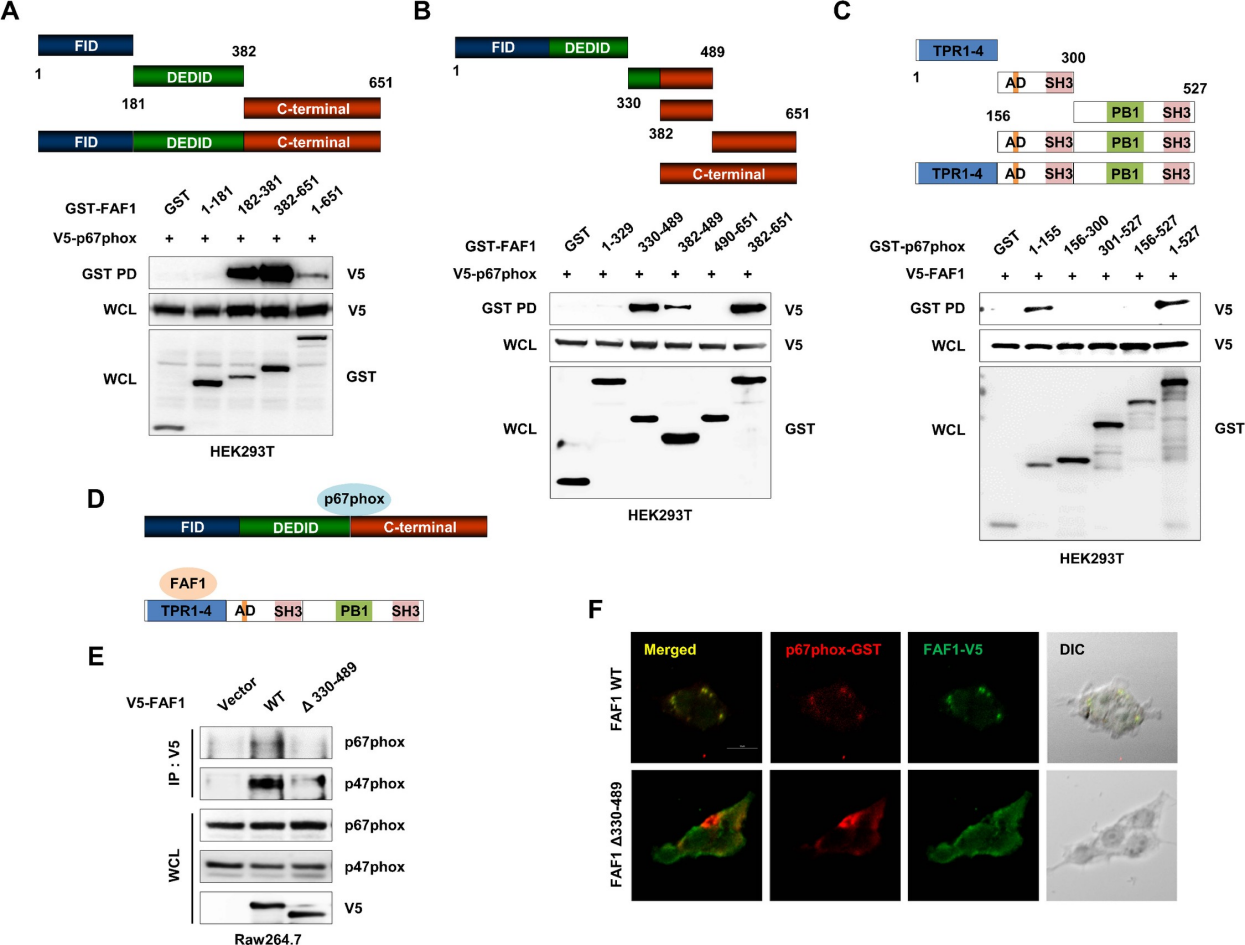
Amino acids 330–489 region of FAF1 is responsible for interaction with p67phox. (A) HEK293T cells were transfected with mammalian GST or GST-tagged FAF1 domain constructs together with V5-tagged p67phox, and at 48hrs, cell lysates were used for GST pull-down assay, followed by immunoblotting with an anti-V5 antibody. (B) HEK293T cells transfected with GST-FAF1 narrow-down domain constructs and V5-tagged p67phox were used for GST pull-down assay. (C) HEK293T cells were transfected with mammalian GST or GST-tagged p67phox domain constructs together with V5-tagged FAF1, and at 48hrs, cell lysates were used for GST pull-down assay, followed by immunoblotting with anti-V5 antibody. (D) Binding mapping. Schematic diagram of FAF1 and p67phox. (E) Raw264.7 cells containing empty vector or V5-tagged FAF1 wild-type or FAF1 Δ330–489 were used for IP with anti-V5 antibody, followed by immunoblotting with anti-p67phox and p47phox antibodies. (F) HEK293T cells were transfected with V5-tagged FAF1 wild-type or V5-tagged FAF1 Δ330–489 together with GST-p67phox, followed by confocal microscopy assay with anti-V5 (green) and GST (red) antibodies. Data are representative of two independent experiments.

FAF1 contains three well characterized domains, the FID, DEDID, and C-terminal domains [21, 24, 29]. To define which domain of FAF1 interacts with p67phox, we generated GST-tagged domain constructs and performed GST pull-down assays in HEK293T cells. p67phox bound strongly to both the DEDID and C-terminal domains of FAF1 (Fig 4, panel A). The region of FAF1 responsible for interaction with p67phox was narrowed down to amino acids 330–489 (Fig 4, panel B). In the reverse experiment, GST-tagged domain constructs of p67phox were generated, and GST pull-down assays were performed in HEK293T cells to determine which domain of p67phox interacts with FAF1. FAF1 bound to the 4X tetratricopeptide repeat (TPR) domain of p67phox (Fig 4, panel C). A diagram of the domains mediating binding between FAF1 and p67phox is shown in panel D (Fig 4, panel D). Next, a mutant construct of FAF1 with a deletion in amino acids 330–489 was generated (Δ330–489). This mutant showed weak binding to endogenous p67phox and p47phox compared with wild-type FAF1 by immunoprecipitation with an anti-V5 antibody in Raw264.7 cells (Fig 4, panel E). Furthermore, ectopically expressed p67phox co-localized with wild-type FAF1 but not FAF1 Δ330–489 in HEK293T cells (Fig 4, panel F). Taken together, these data indicate that amino acids 330–489 of FAF1 comprise the critical region that mediates interaction with the 4X TPR domain of p67phox.

### FAF1 potentiates ROS-mediated inflammatory responses in a p67phox binding-dependent manner

**Fig 5:**
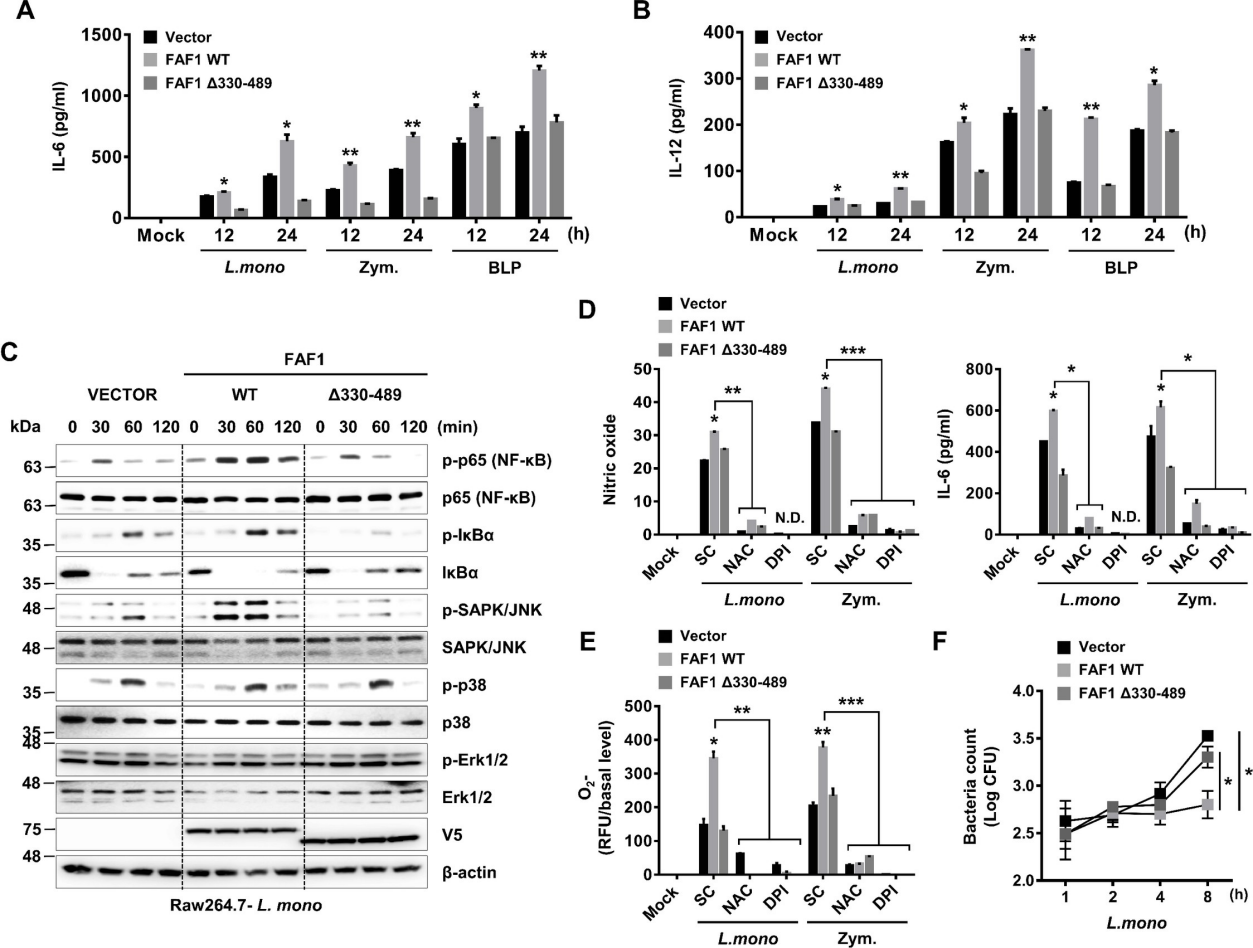
Deletion of amino acids 330–489 in FAF1 attenuates ROS generation and inflammatory responses against *L*. *monocytogenes* infection. (A and B) Raw264.7 cells containing empty vector or V5-tagged FAF1 wild-type or FAF1 Δ330–489 were infected with *L*. *monocytogenes* (MOI = 1) or treated with zymosan (100μg/ml) or BLP (100ng/ml) for the indicated times, and measured for levels of IL-6 (A) and IL-12 (B) by ELISA. (C) Raw264.7 cells containing empty vector or V5-tagged FAF1 wild-type or FAF1 Δ330–489 were infected with *L*. *monocytogenes* (MOI = 1) for the indicated times, and then subjected to immunoblotting with the phosphorylated and total forms of p65, IκBα, JNK, p38, p42/p44 MAPK, V5 or β-actin. (D and E) Raw264.7 cells containing empty vector or V5-tagged FAF1 wild-type or FAF1 Δ330–489 were infected with *L*. *monocytogenes* (MOI = 1) or treated with zymosan w/ or w/o NAC or DPI. At 18hrs, the supernatants were analyzed for NO production or IL-6 secretion (D). At 30min, O_2_- production was determined by DHE red fluorescent dye (E). (F) Raw264.7 cells containing empty vector or V5-tagged FAF1 wild-type or FAF1 Δ330–489 were infected with *L*. *monocytogenes* (MOI = 0.1) for the indicated times, and washed with PBS. Then, cells were lysed by Triton X-100, and intracellular bacteria were determined by CFU titration. Data are representative of two (C-F) or three (A, B) independent experiments. Error bars, mean ± SD. * P < 0.05, ** P < 0.01, *** P < 0.001 (Student`s *t*-test), compared to cells expressing empty vector or FAF1 Δ330–489. N.D., no detection.

As described above, we identified the binding site through which FAF1 interacts with p67phox and found that a deletion mutant of this region (FAF1 Δ330–489) was unable to bind to p67phox. To determine whether FAF1 Δ330–489 lost the ability to promote inflammatory responses due to the weak interaction with p67phox, we generated Raw264.7 cells stably overexpressing a control vector, wild-type FAF1, or FAF1 Δ330–489. The expression level of FAF1 in each stable cell line was determined by western blotting (S12 Fig, panel A). The cells were then stimulated with TLR2 ligands, including *L*. *monocytogenes*, zymosan, and BLP, and levels of proinflammatory cytokines and chemokines in the supernatants were measured. As expected, cells expressing FAF1 Δ330–489 showed reduced secretion of proinflammatory cytokines and chemokines in response to TLR2 signaling compared with cells expressing wild-type FAF1 (Fig 5, panels A-B and S12 Fig, panels B-C). We next evaluated the activation of molecules involved in NF-κB and MAPK signaling in *L*. *monocytogenes-*infected Raw264.7 cells. Cells expressing wild-type FAF1 showed enhanced activation of the NF-κB and SAPK/JNK pathways upon *L*. *monocytogenes* infection, as previously observed (Fig 2, panels A-B), while activation of these signaling molecules was not altered in cells overexpressing FAF1 Δ330–489 compared with control cells (Fig 5, panel C). In addition, IL-6, O_2_, and NO production were impaired in cells overexpressing FAF1 Δ330–489 compared with cells overexpressing wild-type FAF1. Cytokine secretion was not induced in the presence of ROS inhibitors, with no significant difference between individual cell lines (Fig 5, panels D-E). While the growth of intracellular bacteria in cells overexpressing wild-type FAF1 was lower than in control cells, cells overexpressing FAF1 Δ330–489 exhibited a similar level of bacterial growth as control cells (Fig 5, panel F). These findings indicate that the effects of FAF1 on inflammatory responses, and intracellular bacterial clearance through ROS generation are dependent on its interaction with p67phox.

### FAF1 promotes NADPH oxidase activity via stabilization of p67phox

**Fig 6:**
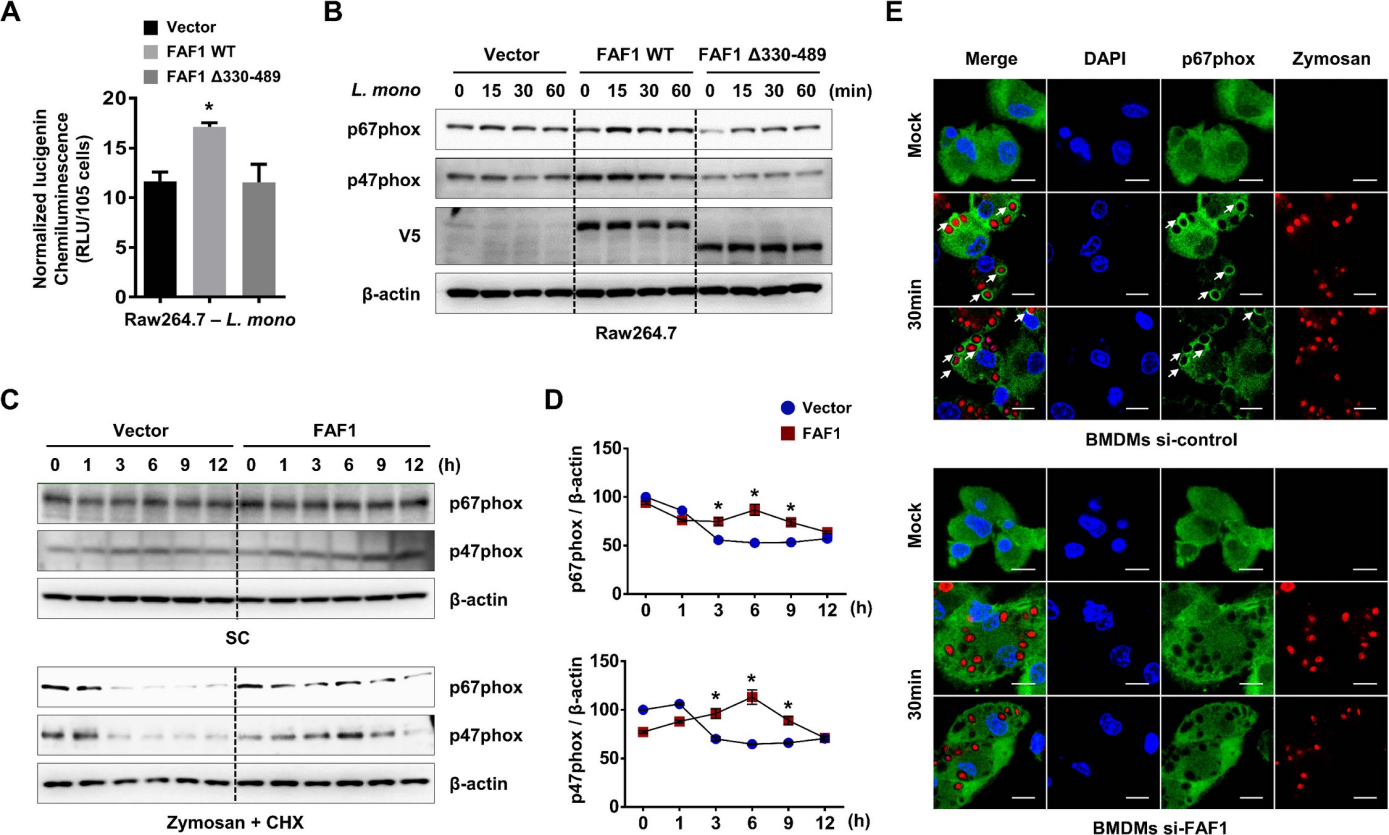
FAF1 promotes the stability of p67phox, leading to increased phagocytic NADPH oxidase activity against *L*. *monocytogenes* infection. (A) Raw264.7 cells containing empty vector or V5-tagged FAF1 wild-type or FAF1 Δ330–489 were analyzed for NADPH oxidase activity upon *L*. *monocytogenes* infection (MOI = 1). (B) Raw264.7 cells containing empty vector or V5-tagged FAF1 wild-type or FAF1 Δ330–489 were infected with *L*. *monocytogenes* for the indicated times, followed by immunoblotting with anti-p67phox, p47phox, V5 and β-actin antibodies. (C) Raw264.7 cells containing empty vector or V5-tagged FAF1 were treated with solvent control (SC, top) or zymosan / cycloheximide (CHX, 0.1μg/ml, bottom) for the indicated times and cell lysates were used for immunoblotting with anti-p67phox, p47phox and β-actin antibodies. (D) The relative expression levels of p67phox or p47phox were shown in graphs. (E) At 36hr post-transfection with siRNA-control or siRNA-FAF1, BMDMs were treated with pHrodo Red zymosan A bioparticles conjugates (0.5mg/ml) for 30min, followed by confocal microscopy with an anti-p67phox antibody. Scale bars represent 10μm. Data are representative of two (C) or three (A, B, E) independent experiments. Error bars, mean ± SD. * P < 0.05 (Student`s *t*-test).

Among NADPH oxidase regulatory proteins, p67phox has a critical role in the activation of NADPH oxidase [4, 34]. To determine whether FAF1 affects phagocytic NADPH oxidase activity, Raw264.7 cells overexpressing control vector, wild-type FAF1, or FAF1 Δ330–489 were infected with *L*. *monocytogenes*, and a chemiluminescence assay was performed to measure NADPH oxidase activity. The result showed that overexpression of wild-type FAF1 augments the phagocytic NADPH oxidase activity, whereas overexpression of FAF1 Δ330–489 doesn`t (Fig 6, panel A). This finding suggested that amino acids 330–489 is the critical region for the interaction with p67phox, which results in increased ROS production in response to *L*. *monocytogenes* infection. We also found that expression of FAF1 leads to higher and persistent expression of p67phox in a binding-specific manner (Fig 6, panel B). Overexpression of FAF1 induces a bit more mRNA expression of p67phox and p47phox without stimulation (S13 Fig). Additionally, Raw264.7 cells overexpressing control vector, wild-type FAF1 were treated with mock or zymosan/cycloheximide and used for immunoblot analysis for expression levels of p67phox and p47phox over time. This result showed that overexpression of FAF1 increases the stability of p67phox and p47phox upon zymosan treatment (Fig 6, panel C). The intensity of the protein bands on the blot shown in Fig 6, panel D is quantitated (Fig 6, panel D). Furthermore, to check the localization of p67phox around phagosomes depending on expression level of FAF1, BMDMs were treated with zymosan particles for 30 min following siRNA-mediated knockdown of FAF1, then followed by confocal microscopy using anti-p67phox antibody (Fig 6, panel E). As correlated with prior data, knockdown of FAF1 exhibited considerable decrease of p67phox localized to phagosomal membranes compared to control, which supports evidently that FAF1 potentiates the stability of p67phox in phagosomes. Collectively, these results suggest that FAF1 in macrophages effectively augments p67phox stability in response to infection with *L*. *monocytogenes*, resulting in increased phagocytosis-mediated NADPH oxidase activity.

## Discussion

FAF1 (Fas-associated factor 1), a member of the Fas death-inducing signal complex, modulates a variety of biological processes by interacting with diverse molecules [24–26]. However, the role of FAF1 in host defense against bacterial infection remains unclear. Here, we report that FAF1 is a positive regulator that increases activity of the phagocytic NADPH oxidase complex, resulting in production of ROS and in activation of NF-κB signaling, inflammatory responses, and antibacterial activity upon *L*. *monocytogenes* infection. First, FAF1^gt/gt^ mice exhibited reduced serum cytokine levels, reduced inflammatory gene expression, and increased bacterial burden during *L*. *monocytogenes* infection. Second, primary macrophages (BMDMs and PMs) isolated from FAF1^gt/gt^ mice showed decreased ROS production and inflammatory responses as well as bacterial clearance than macrophages from FAF1^+/+^ mice upon *L*. *monocytogenes* infection or TLR2 stimulation. Consistent with these data, knockdown of FAF1 in Raw264.7 cells also showed significantly reduced ROS production, NF-κB activation and inflammatory responses, upon *L*. *monocytogenes* infection. Third, FAF1 transiently interacted strongly with the p67phox-p47phox-p40phox complex at early time points after *L*. *monocytogenes* infection in macrophages, and FAF1 region comprising amino acids 330–489 was responsible for interaction with the TPR domain of p67phox. Finally, interaction between FAF1 and p67phox stabilized p67phox and increased activity of phagocytic NADPH oxidase upon *L*. *monocytogenes* infection. Collectively, these findings strongly suggest that FAF1 plays a crucial role in promoting antibacterial responses by interacting with p67phox in macrophages during intracellular microbial infection.

NADPH oxidase and dual oxidase induce ROS production by various cells and tissues in response to growth factors, cytokines, and pathogen-mediated signals [1]. Among these, the phagocyte NADPH oxidase is a multi-component complex in which the membrane glycoprotein gp91phox (known as NOX2) is tightly associated with p22phox; this complex is activated via association with cytosolic regulatory proteins such as p67phox, p47phox, p40phox, and the small GTPase Rac, resulting in ROS generation [1, 3, 4]. Based on homology to gp91phox (Nox2), the Nox family of NADPH enzymes comprises seven members: Nox1 through Nox5, plus Duox1 and Duox2 [35]. All gp91phox-related enzymes (except gp91phox (Nox2)) belonging to the Nox family are non-phagocytic enzymes expressed in epithelial or endothelial cells within diverse tissues and organs [1]. ROS generated by these NADPH oxidases take part in biological processes such as cell signaling, hormone biosynthesis, and host innate immune responses [5, 36]. In particular, ROS play essential roles in phagocyte-mediated defense against bacterial infection; ROS kill engulfed pathogens directly, or indirectly by activating intracellular signaling pathways related to inflammatory responses, which then protect the host. ROS are necessary to eliminate intracellular bacteria such as *mycobacteria*, *Listeria*, *and Salmonella*, efficiently [37].

Recognition of pathogens by TLRs is the first line of host innate defense; indeed, interaction between TLRs and NADPH oxidase in phagocytes has been well studied. For example, Yang et.al., report that Nox2 is essential for TLR2-dependent inflammatory responses and for intracellular control during mycobacterial infection. They showed that Nox2 and TLR2 interact directly during mycobacterial infection [13, 14]. Moreover, the interplay between TLR4 and NADPH oxidases such as Nox4 or Nox1 was studied in non-phagocytic cells [16, 38]. However, lipopolysaccharide (LPS; a TLR4 agonist) also activates NADPH oxidase in phagocytes indirectly by increasing association of gp91phox (Nox2) with regulatory proteins in plasma membrane [39]. Here, we demonstrated that FAF1 is a crucial regulator of the phagocytic NADPH oxidase (Nox2) complex required for ROS production by macrophages in response to *L*. *monocytogenes* infection as well as TLR2 stimulation. We also examined whether FAF1 regulates inflammatory responses in macrophages stimulated by TLR4. As results, TLR4-mediated cytokine secretion by BMDMs isolated from FAF1^gt/gt^ mice was lower than that by cells from FAF1^+/+^ mice (S14 Fig, panels A-B). These results suggest that FAF1 also activates signaling pathways related to TLR4 stimulation, as proposed by DeLeo et.al. However, further studies demonstrating a detailed mechanism of how FAF1 controls TLR4 signaling pathway are needed.

Nox2 in resting phagocytes is inactive; however, it is activated by phagocytosis of invading bacteria, leading to ROS production and their subsequent effects on host defense (e.g., killing bacteria and regulating intracellular signaling). Once bacteria are recognized by host TLRs, NADPH oxidase Nox2 (gp91phox) heterodimerizes with p22phox at the phagocyte membrane and is rapidly activated by cytosolic regulatory proteins [1, 3, 4]. These p22phox and cytosolic regulatory proteins (p67phox, p47phox, p40phox, and the small GTPase Rac) are indispensable for regulation of NADPH oxidase; indeed, increasing evidence suggests that it is important to control NADPH oxidase subunits to ensure appropriate ROS generation. For example, RUBICON interacts with p22phox, thereby increasing ROS production in response to infection by Gram-positive bacteria [12]. Moreover, Nox-dependent ROS production occurs in Parkinson’s disease (autosomal recessive, early onset) 7 (Park7)-p47phox [40]. Our findings suggest that FAF1 is a key molecule that regulates activation of NADPH oxidase via strong interaction with p67phox.

As noted above, mass spectrometry analysis identified the NoxA1 protein as an interacting protein of FAF1. NoxA1 is expressed at high levels by colon epithelial cells and is responsible for activation of Nox1 (and thereby for subsequent ROS production) [1, 3, 33]. p67phox is a homolog of NoxA1 expressed by phagocytes. Thus, we hypothesized that FAF1 interacts with p67phox to regulate ROS generation in macrophages. In this study, we found that FAF1 interacts with p67phox at an early time point after *L*. *monocytogenes* infection. Moreover, amino acids 330–489 of FAF1 are required for interaction with p67phox. Conversely, FAF1 interacted with the TPR domain (amino acids 1–155) of p67phox. This domain, which comprises four 34 amino acid-long TPR motifs, is involved in a variety of protein-protein interactions [4, 32, 41, 42]. In macrophage cell lines stably overexpressing wild-type FAF1 or FAF1 Δ330–489, we found that overexpression of wild-type FAF1 but not FAF1 Δ330–489 increased NADPH oxidase activity, ROS production, proinflammatory cytokine production, and antimicrobial activity. Ultimately, interaction between FAF1 and p67phox facilitated stabilization of p67phox and increased activity of NADPH oxidase upon *L*. *monocytogenes* infection. These results suggest that regulation of phagocytic NADPH oxidase by FAF1 is dependent on binding to p67phox. Consequently, we demonstrated that FAF1 positively regulates the NADPH oxidase 2 complex via stabilization of p67phox.

FAF1 interacts potentially with many different proteins and functions as a negative and/or positive regulator in a variety of biological possesses [25, 26, 43]. Previous studies report that FAF1 homologs suppress antibacterial immunity in *Drosophila* and *Locusta migratoria* [44, 45]. In addition, Park et al. showed that FAF1 suppresses IKK activation and nuclear translocation of NF-κB in fibroblasts [29, 46]. However, we clearly demonstrate that FAF1 acts as a positive regulator of antibacterial responses by regulating ROS production. In particular, we identified the physiological role of FAF1 in defense responses against *L*. *monocytogenes* infection in FAF1^+/+^ and FAF1^gt/gt^ mice. FAF1^gt/gt^ mice attenuates bacterial clearance due to reduced inflammatory responses. We also have focused on the role of FAF1 in phagocytes such as macrophages *in vitro*. Raw264.7 cells in which FAF1 was knocked down, as well as BMDMs and resident PMs isolated from FAF1^gt/gt^ mice, showed lower ROS production, proinflammatory responses, and bacterial killing activity than FAF1^+/+^ mice upon *L*. *monocytogenes* infection. In addition, activation of molecules involved in NF-κB signaling was markedly reduced in FAF1-knockdown cells when exposed to *L*. *monocytogenes*. However, the upstream molecules responsible for FAF1 activation upon *L*. *monocytogenes* infection or TLR2 stimulation remain unclear. To assess this, further studies are necessary to examine the detailed molecular mechanisms (e.g., phosphorylation of FAF1) that operate in macrophages under infectious conditions.

In summary, we demonstrated that FAF1 is a critical positive regulator of the phagocytic NADPH oxidase (Nox2) complex responsible for ROS generation by phagocytes upon *L*. *monocytogenes* infection or TLR2 stimulation. FAF1 interacts directly with p67phox and stabilizes p67phox, thereby triggering NADPH oxidase-mediated ROS production, release of proinflammatory cytokines, and bacterial clearance in response to infection by *L*. *monocytogenes*. Taken together, the results suggest a plausible mechanism involving interaction between p67phox and FAF1 and increases our understanding of molecules that control ROS signaling and antibacterial defense responses against *L*. *monocytogenes* infection or TLR2 stimulation.

## Materials and methods

### Ethics statement

All animal experiments were managed in strict accordance with the Guide for the Care and Use of Laboratory Animals (National Research Council, 2011) and performed in BSL-2 and BSL-3 laboratory facilities with the approval of the Institutional Animal Care and Use Committee of Bioleaders Corporation (Reference No., BLS-ABSL-16-002) and Chungnam National University (Reference No., CNU-00763).

### Reagents and antibodies

Zymosan (tlrl-zyn), Pam3CSK4 (tlrl-pms) were purchased from Invivogen. DPI(D2926), NAC(A9165), Cycloheximide(C7698), DHE(D7008), Amplex red and peroxidase were purchased from Sigma. DCFH-DA and pHrodo Red Zymosan A Bioparticles conjugate (P35364) were obtained from Molecular probe. For western blot analysis, specific antibodies for p-IKKα/β (2697), IKKβ (8943), p-JNK (4668), JNK (9258), p-NF-κB p65 (3033), NF-κB p65 (4764), p-Erk1/2 (9101), Erk1/2 (9102), p-p38 (4631), p38 (9212), p-IκBα (2859), IκBα (4812) and p67phox (3923) were purchased from Cell Signaling Technology. Antibodies for FAF1 (sc-393965), p47phox (sc-14015), IKKα (sc-7606), β-actin (sc-47778), GST (sc-138) were purchased from Santa Cruz Biotechnology. Anti-p67phox antibody (ab109366) was purchased from abcam. Anti-V5 antibody (46–0705) was purchased from Invitrogen life technology. The anti-FAF1 monoclonal antibody was kindly provided by Dr. Eunhee Kim (Chungnam National University, Daejeon, Korea).

### Bacterial strain

*L*.*monocytogenes* (KVCC-BA0000087) was from Korean Veterinary Culture Collection (KVCC), and grown at 37°C in Brain Heart Infusion (BHI) broth medium (BD 237500). Log phase bacteria (O.D. value, 0.6–0.8) were used for all assay. Cultures were aliquoted and stored at -80°C. To determine bacterial titer (Colony-forming unit, CFU), bacteria thawed was diluted 10-fold. Each diluent was plated on BHI agar (BD 241830) and incubated at 37°C for one day.

### Mice and *in vivo* experiments

FAF1^+/+^ and FAF1^gt/gt^ mice on a C57BL/6 background were kindly provided by Dr. Eunhee Kim (Chungnam National University, Daejeon, Korea). A hypomorphic allele, designated FAF1^gt/gt^, was generated by a gene-trap insertion in intron 8 [47]. All mice were bred in pathogen-free condition. Offspring were genotyped by PCR as previously described [27].

Sex-matched mice (six-week-old) were intraperitoneally infected with *L*. *monocytogenes* (5 × 10^5^ CFU/mouse). Liver, spleen, peritoneal macrophage and serum were collected to determine the bacterial load, cytokines or chemokines levels or mRNA levels of cytokines or chemokines as described below.

### Construction of expression plasmids

FAF1 plasmid was kindly provided by Dr. Eunhee Kim (Chungnam National University, Daejeon, Korea). To generate plasmid constructs with GST expressing vector, FAF1 and its mutants or p67phox and its mutants were amplified by PCR and inserted into pEBG vector. For construction of V5-tagged expression plasmid, FAF1, FAF1 Δ330–489 and p67phox were amplified by PCR and inserted into pIRES-V5.

### Cell preparation, cell culture and transfection

Raw264.7 cells, HEK293T cells, BMDMs and PMs were maintained in Dulbecco’s Modified Eagle’s medium (Gibco) supplemented with 10% FBS (Thermo-Hyclone) and antibiotic-antimycotic (Gibco) at 37°C in 5% CO_2_. To establish stable expressing cell line, Raw264.7 cells were transfected with empty vector or V5-tagged FAF1 wild-type or V5-tagged FAF1 Δ330–489 using Lipofectamine 2000 (Invitrogen), then selected with 2μg/ml puromycin (Gibco) containing culture media for 2 weeks. To generate FAF1 knockdown cell lines, Raw264.7 were infected with lentivirus containing non-specific shRNA or FAF1-specific shRNA in the presence of 8μg/ml polybrene (Sigma AL118), and then selected with 2μg/ml puromycin for 2 weeks as previously described [27]. Resident peritoneal macrophages were obtained by flushing peritoneal cavity of FAF1^+/+^ and FAF1^gt/gt^ mice with HBSS w/o phenol red as previously described [48]. BMDMs were isolated from FAF1^+/+^ or FAF1^gt/gt^ mice and cultured for 6 days in medium containing 20 ng/ml recombinant mGM-CSF (Creagen) as previously described [27].

### Lentiviral short hairpin RNAs (shRNA)

For silencing of FAF1 gene expression, the pGIPZ lentiviral vector, which contains FAF1-specific shRNA sequences was purchased from Open Biosystems. (http://www.openbiosystems.com). Lentiviruses were produced as previously described [27]. In brief, HEK293T cells were transiently transfected with packaging plasmids (psPAX2 and pMD2.VSV-G) and pGIPZ containing non-specific shRNA or FAF1-specific shRNA sequences using Lipofectamine 2000. At 48–72 hr post-transfection, virus-containing media was collected and filtrated (0.45 μm filter, Millipore).

### Small interfering RNA (siRNA) transfection

The sequence of the mouse FAF1-specific siRNA #1 (duplex) were as follow, 5`-CCG CCU UCA UCA UCC AGC C-3`and 5`-GGC UGG AUG AUG AAG GCG G-3`. The mouse FAF1-specific siRNA duplex #2–4 (14084–1, 14084–2, and 14084–3) were purchased from Bioneer Corp. The mouse p47phox-specific siRNA (sc-36157) was purchased from Santa Cruz Biotechnology. A non-targeting siRNA was used as a control. Cells were transfected with duplex siRNA using Lipofectamine RNAiMAX (Invitrogen), according to the manufacturer`s protocol, then incubated for 36 hrs before stimulation.

### Immunoprecipitation and GST pull-down assay

For immunoprecipitation, Raw264.7 cells or BMDMs were lysed with RIPA buffer containing protease inhibitor cocktail (Roche). Lysates were incubated with a primary antibody overnight at 4°C, followed by incubation with protein A/G agarose (Santa Cruz Biotechnology) for 3hrs at 4°C. Then immunoprecipitates were washed with 1% nonidet P-40 for 3 times. For GST-pull down assay, HEK293T cells were transiently transfected with the indicated plasmids, and at 36hr post-transfection, were lysed with RIPA buffer containing protease inhibitor cocktail (Roche). Lysates were pre-cleared by sepharose 6B (GE Healthcare) for 3hrs at 4°C, followed by incubation with glutathione (GSH) sepharose 4B (GE Healthcare) overnight at 4°C. Precipitated beads were washed with 1% nonidet P-40 for 3 times.

### Immunoblotting

Whole cell lysates (WCLs) were prepared using Radio-immunoprecipitation assay (RIPA) lysis buffer (50mM Tris-HCl, 150mM NaCl, 0.5% sodium deoxycholate, 1% IGEPAL, 1mM NaF, 1mM Na_3_VO_4_, proteinase inhibitor cocktail). Samples were separated by SDS-PAGE and transferred onto a PVDF membrane (Bio-Rad) using Trans-Blot semi dry transfer cell (Bio-Rad). Membranes were blocked for 1hr in 5% bovine-serum albumin with TBST or 5% skim milk within PBST, followed by incubation with primary antibody overnight at 4°C. Next day, membranes were washed with TBST or PBST, and incubated at room temperature with HRP-conjugated secondary antibody. Then, membranes were washed 3 times with TBST or PBST, followed by developing with Western blotting detection reagents (GE healthcare, ECL select Western Blotting Detection Reagent).

### Mass spectrometry analysis

HEK293 cells were lysed with RIPA buffer containing protease inhibitor cocktail (Roche). Lysates were incubated with anti-FAF1 antibody overnight at 4°C, followed by incubation with protein A/G agarose (Santa Cruz Biotechnology) for 3hrs at 4°C. Then immunoprecipitates were washed with 1% nonidet P-40 for 3 times and separated by PAGE gels, followed by Coomassie blue staining. Protein bands were excised from the gel and identified by Q-TOF mass spectrometer.

### ELISA

ELISA was performed to detect the secreted cytokines and chemokines in sera or cell culture supernatants. Mouse IL-6 (BD biosciences, 555240) and mouse IL-12 (BD biosciences, 555165), RANTES (Invitrogen), MCP-1 (Invitrogen) were used for analysis according to the manufacturer’s protocols.

### Measurement of intracellular ROS and NO and determination of NADPH oxidase activity

The oxidative fluorescent dye 20μM CM-H2DCFDA (Molecular probe), 20uM Amplex red (Sigma) with 0.1 unit/ml peroxidase (Sigma) and 20μM DHE (Sigma) were used to detect intracellular total ROS (Excitation 485nm, Emission 530nm), H_2_O_2_ production (Excitation 530nm, Emission 620nm) and O_2_- (Excitation 485nm, Emission 620nm), respectively, using fluorescence module of GloMax-Multi Microplate Reader (Promega, E7031). NO detection in culture media was performed using Griess reagent (G4410, Sigma) at 540 nm. Lucigenin (M8010) and NADPH (N1630) were used to determine NADPH oxidase activity as previously described [12].

### Quantitative real time PCR

Total RNA was isolated from cells and murine tissues using the RNeasy Mini Kit (Qiagen). cDNA synthesis was performed using reverse transcriptase (TOYOBO), cDNA was quantified by real-time polymerase chain reaction (PCR) using QuantiTect SYBR Green PCR kit (TOYOBO), according to the manufacturer`s instructions on a Rotorgene (Qiagen). The primer sequences were as follow: mFAF1, 5'-GGT GAC TGC CAT CCT GTA TTT T-3' (forward) and 5'-TGC TCT GTT GGT GTC CTT TG-3`(reverse), mGAPDH, 5`-TGA CCA CAG TCC ATG CCA T-3`(forward) and 5`- GAC GGA CAC ATT GGG GGT AG-3`(reverse); mIL-6, 5`- GAC AAC TTT GGC ATT GTG G-3`(forward) and 5`- ATG CAG GGA TGA TGT TCT G-3`(reverse); mIL-12p40, 5'-CAG AAG CTA ACC ATC TCC TGG TTT G-3' (forward) and 5'-TCC GGA GTA ATT TGG TGC TTC ACA C-3' (reverse); mIL-1β, 5`-TTG TGG CTG TGG AGA AGC TGT-3`(forward) and 5`-AAC GTC ACA CAC CAG CAG GTT-3`(reverse); mCOX-2, 5`-TGA GTA CCG CAA ACG CTT CT-3`(forward) and 5`-CTC CCC AAA GAT AGC ATC TGG-3`(reverse); miNOS, 5`-TGG GAA TGG AGA CTG TCC CAG-3`(forward) and 5`-GGG ATC TGA ATG TGA TGT TTG-3`(reverse); CXCL10, 5`-GCC GTC ATT TTC TGC CTC A-3`(forward) and 5`-CGT CCT TGC GAG AGG GAT C-3`(reverse); RANTES, 5`- CCA GAG AAG AAG TGG GTT CAA G-3`(forward) and 5`- AAG CTG GCT AGG ACT AGA GCA A-3`(reverse); mp67phox, 5´-CAG ACC CAA AAC CCC AGA AA-3´ (forward) and 5´-AGGGTGAATCCGAAGCTCAA-3´ (reverse); mp47phox, 5´-GTC CCT GCA TCC TAT CTG GA-3´ (forward) and 5´-TAT CTC CTC CCC AGC CTT CT-3´ (reverse); mgp91phox, 5´-TCG CTG GAA ACC CTC CTA TG-3´ (forward) and 5´-GGA TAC CTT GGG GCA CTT GA-3´ (reverse) (Bioneer, Daejeon, Republic of Korea).

### Confocal microscopy

BMDMs or HEK293T cells were seeded into eight-chamber slides, followed by treatment or transfection desired. The cells were fixed in 4% paraformaldehyde at RT for 20min, then permeabilized by incubation for 20min with 100% Methanol at -20°C. Then, the fixed cells were incubated with 2% FBS for 1hr to block non-specific binding of antibodies. The appropriate primary antibodies were incubated overnight at 4°C. The cells were washed three times with PBST, and incubated with the appropriate secondary antibodies (Invitrogen) for 1hr at RT without light exposure. Then, the cells were washed three times with PBST, and stained with DAPI (ratio, 1:100,000), washed with PBS, and mounted in mounting solution (VECTOR). Images were acquired under a Nikon laser scanning confocal microscope (C2plus) and analyzed using NIS-Elements software.

### Quantification of intracellular bacteria growth

BMDMs or Raw264.7 cells were infected with *L*. *monocytogenes* (MOI = 0.1) for 1hr and washed 3 times with sterile PBS, followed by incubation with DMEM containing gentamycin (100μg/ml) for the indicated times. Finally, cells were harvested and lysed with 0.1% Triton X-100 (Sigma) to release the intracellular bacteria, and cell lysates were then diluted 10-fold in BHI broth (BD). Each sample were plated on BHI agar (BD) and incubated at 37°C for one day. Colony-forming unit (CFU) was utilized to ensure quantification of intracellular bacteria.

### Graphing and statistics

Prism 6 software (GraphPad Software) was used for charts and statistical analyses. The significance of results was analyzed by an unpaired two-tailed Student’s *t*-test and Mann-Whitney test with a cutoff *P* value of 0.05. Error bars and *P*-values are indicated in the figure legends.

## Supporting information

S1 Fig PMs isolated from FAF1^gt/gt^ mice reduce proinflammatory gene expression in response to *L. monocytogenes* infection. FAF1^+/+^ and FAF1^gt/gt^ mice were intraperitoneally challenged with *L*. *monocytogenes* (5.0 × 10^5^ CFU/mouse). At 24h post-infection, elicited PMs were isolated from abdominal cavity, and used for measuring mRNA level of cytokines by quantitative real-time PCR. The value was normalized by wild type level in each pooled group. Data show the means ± SD of values of two independent experiments of which cells were obtained from 6 mice. * P < 0.05, ** P < 0.01 (Mann-Whitney test).
(TIF)

S2 Fig FAF1 gene is significantly induced by *L. monocytogenes* infection in macrophages. (A) Raw264.7 cells were infected with *L*. *monocytogenes* (MOI = 0.1) for the indicated times, followed by quantitative real-time PCR analysis to estimate FAF1 mRNA expression level. (B and C) BMDMs (B) and Raw264.7 cells (C) were infected with *L*. *monocytogenes* (MOI = 3) for 2hrs and harvested at indicated times, followed by quantitative real-time PCR analysis to estimate FAF1 mRNA expression level. Data (A) show the mean ± SD of values of two independent experiments. Data (B, C) are representative of two independent experiments. * P < 0.05, ** *P* < 0.01 (Student`s *t*-test).
(TIF)

S3 Fig PMs isolated from FAF1^gt/gt^ mice reduce proinflammatory responses upon *L. monocytogenes* infection or TLR2 stimulation. (A) BMDMs were isolated from FAF1^+/+^ and FAF1^gt/gt^ mice, followed by IB with anti-FAF1 or β-actin antibody. (B) Resident PMs were isolated from FAF1^+/+^ and FAF1^gt/gt^ mice, followed by IB with anti-FAF1 or β-actin antibody. (C and D) FAF1^+/+^ and FAF1^gt/gt^ PMs were stimulated with *L*. *monocytogenes* (MOI = 1) or zymosan (100μg/ml) or BLP (100ng/ml). At the indicated time, supernatants were harvested and measured for IL-6 (C) or IL-12 (D) secretion by ELISA. Data are representative of two (C, D) independent experiments. Error bars, mean ± SD. * P < 0.05, ** P < 0.01 (Student`s *t*-test).
(TIF)

S4 Fig FAF1 knockdown Raw264.7 cells show reduced chemokines during *L. monocytogenes* infection. (A) Control Raw264.7 cells (shRNA-NS) and FAF1 knockdown Raw264.7 cells (shRNA-FAF1) were used for immunoblotting with anti-FAF1 or β-actin antibody. (B and C) Control Raw264.7 cells (shRNA-NS) and FAF1 knockdown Raw264.7 cells (shRNA-FAF1) were infected with *L*. *monocytogenes* (MOI = 1). At the indicated time, supernatants were harvested and measured for RANTES (B), or MCP-1 (C) secretion by ELISA. Data are representative of two independent experiments. Error bars, mean ± SD. * P < 0.05, ** P < 0.01 (Student`s *t*-test).
(TIF)

S5 Fig FAF1^gt/gt^ PMs show reduced proinflammatory genes during *L. monocytogenes* infection. FAF1^+/+^ and FAF1^gt/gt^ PMs were infected with *L*. *monocytogenes* (MOI = 1) for 2hrs. At 12hr post-infection, cells were analyzed for IL-6, iNOS, COX-2, CXCL10, and RANTES mRNA expression by quantitative real-time PCR. Data are representative of two independent experiments. Error bars, mean ± SD. * P < 0.05, ** P < 0.01, *** P < 0.001 (Student`s *t*-test).
(TIF)

S6 Fig NoxA1 was identified by mass spectrometry analysis. HEK293 cells were used for immunoprecipitation with anti-FAF1 antibody. The immunoprecipitates were separated by PAGE gel, followed by Coomassie blue staining. Protein bands were excised from the gel and identified by Q-TOF mass spectrometer.
(TIF)

S7 Fig Validation for mouse FAF1-specific siRNA available in Raw264.7 cells and BMDMs. (A) At 36hr post-transfection with a non-targeting control siRNA (si-control) or four mouse FAF1-specific siRNA (si-FAF1) candidates (200nM), Raw264.7 cells were harvested and used for immunoblotting with anti-FAF1 or β-actin antibody. (B) At 36hr post-transfection with a control siRNA or FAF1-specific siRNA #2 (200nM), BMDMs were harvested and used for immunoblotting with anti-FAF1 or β-actin antibody.
(TIF)

S8 Fig Knockdown of FAF1 leads to reduced interaction with p67phox upon *L. monocytogenes* infection in BMDMs and Raw264.7 cells. (A) At 36hr post-transfection with a control siRNA or FAF1-specific siRNA #2 (200nM), BMDMs were infected with *L*. *monocytogenes* for the indicated times, followed by immunoprecipitation with anti-FAF1 antibody. The immunoprecipitates were used for immunoblotting with anti-p67phox, or anti-FAF1 antibody. (B) At 36hr post-transfection with a control siRNA or FAF1-specific siRNA #2 (200nM), Raw264.7 cells were infected with *L*. *monocytogenes* for the indicated times, followed by immunoprecipitation with anti-FAF1 or anti-p67phox antibody. The immunoprecipitates were used for immunoblotting with anti-p67phox or anti-FAF1 antibody. Data are representative of two independent experiments.
(TIF)

S9 Fig Knockdown of FAF1 attenuates ROS production upon zymosan treatment in BMDMs, PMs, and Raw264.7 cells. (A, B) BMDMs (A) or PMs (B) from FAF1^+/+^ or FAF1^gt/gt^ were treated with zymosan (100μg/ml) for the indicated times, then then used for measuring H_2_O_2_ or total ROS production. (C) Control Raw264.7 cells (shRNA-NS) and FAF1 knockdown Raw264.7 cells (shRNA-FAF1) were treated with zymosan for 30min, then used for measuring O_2_- production. Data are representative of two independent experiments. Error bars, mean ± SD. * P < 0.05, ** P < 0.01 (Student`s *t*-test).
(TIF)

S10 Fig FAF1 does not affect IKKα/β phosphorylation upon *L. monocytogenes* infection in Raw264.7 cells. At 36hr post-transfection with a control siRNA or FAF1-specific siRNA #2 (200nM), Raw264.7 cells were infected with *L*. *monocytogenes* for the indicated times, followed by immunoblotting with anti-p-IKKα/β, anti-IKKα, anti-IKKβ, anti-FAF1, or anti-β-actin antibody. Data are representative of two independent experiments.
(TIF)

S11 Fig Overexpression of FAF1 increases inflammatory responses depending on p47phox expression upon *L. monocytogenes* infection in Raw264.7 cells. (A) At 36hr post-transfection with a control siRNA or p47phox-specific siRNA (200nM), Raw264.7 cells containing vector or V5-tagged FAF1 were infected with *L*. *monocytogenes* for the indicated times, followed by immunoblotting with anti-p-p65, anti-p65, anti-p-IκBα, anti-IκBα, anti-p47phox, anti-V5, or β-actin antibody. (B) At 36hr post-transfection with a control siRNA or p47phox-specific siRNA (200nM), Raw264.7 cells containing vector or V5-tagged FAF1 were infected with *L*. *monocytogenes* for 12hrs. The supernatants were used for measuring IL-6 cytokine production using ELISA. Data are representative of two (A) or three (B) independent experiments. Error bars, mean ± SD. * P < 0.05, ** P < 0.01 (Student`s t-test).
(TIF)

S12 Fig Ectopic expression of FAF1 but not FAF1 Δ330–489 increases the chemokine production in Raw264.7 cells upon *L. monocytogenes* infection or TLR2 stimulation. (A) Raw264.7 cells containing vector or V5-tagged FAF1 wild-type or V5-tagged FAF1 Δ330–489 were used for immunoblotting with anti-V5 or β-actin antibody. (B and C) Raw264.7 cells containing vector or V5-tagged FAF1 wild-type or V5-tagged FAF1 Δ330–489 were infected with *L*. *monocytogenes* (MOI = 1, B) or treated with zymosan (100μg/ml, C). At the indicated times, supernatants were harvested and measured for RANTES or MCP-1 levels by ELISA. Data are representative of three independent experiments. Error bars, mean ± SD. * P < 0.05, ** P < 0.01 (Student`s *t*-test).
(TIF)

S13 Fig Ectopic expression of FAF1 but not FAF1 Δ330–489 increases mRNA expression of p67phox, and p47phox not gp91phox without stimulation. Raw264.7 cells containing vector or V5-tagged FAF1 wild-type or V5-tagged FAF1 Δ330–489 were used for quantitative real-time PCR analysis to estimate mRNA expression level of p67phox, p47phox or gp91phox. Data show the mean ± SD of values of two independent experiments. * P < 0.05, ** P < 0.01 (Student`s *t*-test).
(TIF)

S14 Fig FAF1^gt/gt^ BMDMs show lower level of cytokines upon TLR4 stimulation. (A and B) FAF1^+/+^ and FAF1^gt/gt^ BMDMs were stimulated with LPS (100ng/ml) or *S*. *typhimurium* (MOI = 0.1). At the indicated time, supernatants were harvested and measured for IL-6 (A) or IL-12 (B) levels by ELISA. Data show the mean ± SD of values of two independent experiments. * P < 0.05, ** P < 0.01 (Student`s t-test).
(TIF)
