# Supplementary figures and images for: Fas-associated factor 1 mediates NADPH oxidase-induced reactive oxygen species production and proinflammatory responses in macrophages against Listeria infection
Source: PLoS Pathog. 2019 Aug 14;15(8):e1008004. doi: 10.1371/journal.ppat.1008004 (PMC6709923; doi:10.1371/journal.ppat.1008004)

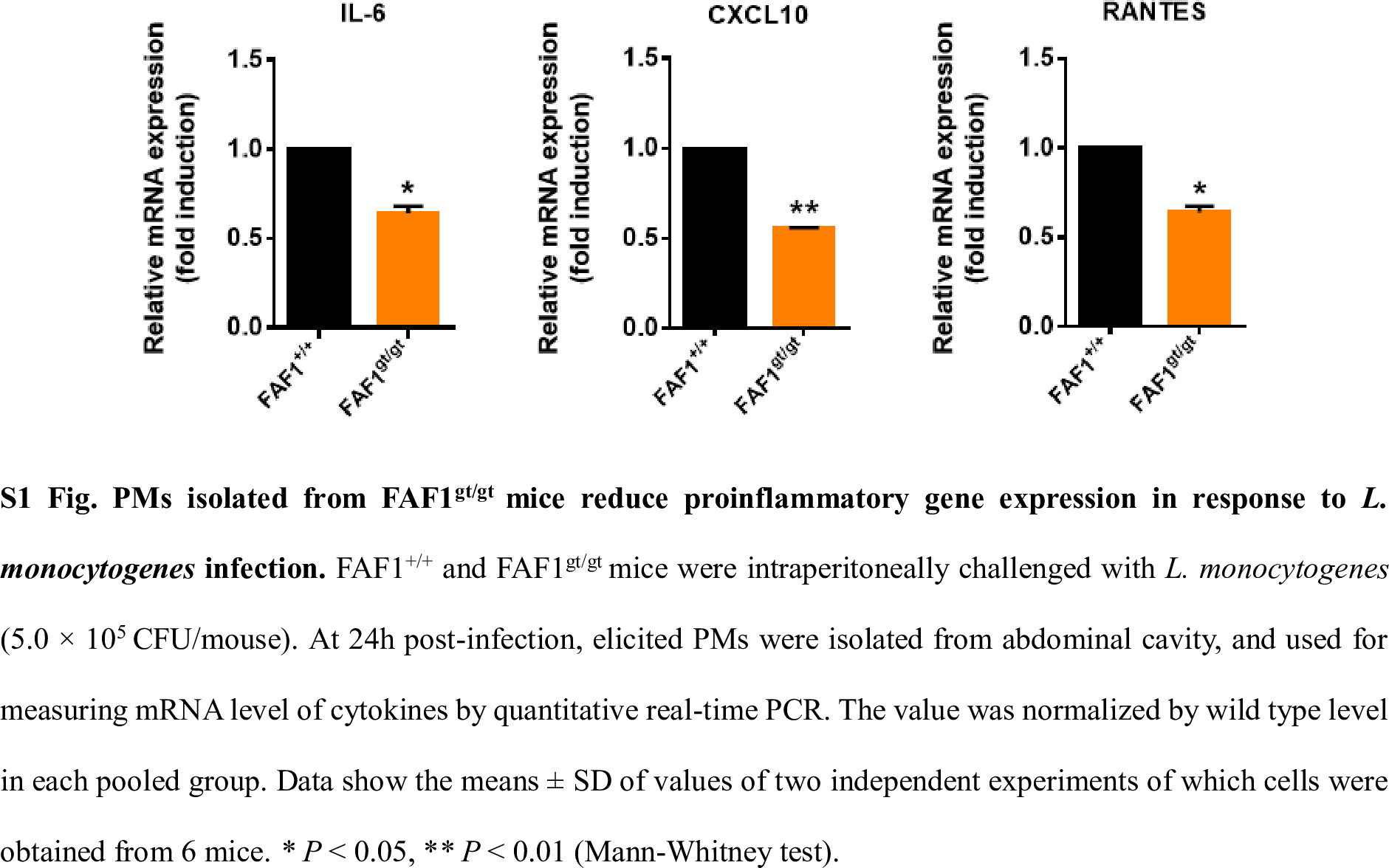

Supplement: S1 Fig — FAF1+/+ and FAF1gt/gt mice were intraperitoneally challenged with L. monocytogenes (5.0 × 10^5^ CFU/mouse). At 24h post-infection, elicited PMs were isolated from abdominal cavity, and used for measuring mRNA level of cytokines by quantitative real-time PCR. The value was normalized by wild type level in each pooled group. Data show the means ± SD of values of two independent experiments of which cells were obtained from 6 mice. * P < 0.05, ** P < 0.01 (Mann-Whitney test). (TIF) [file ppat.1008004.s001.tif]

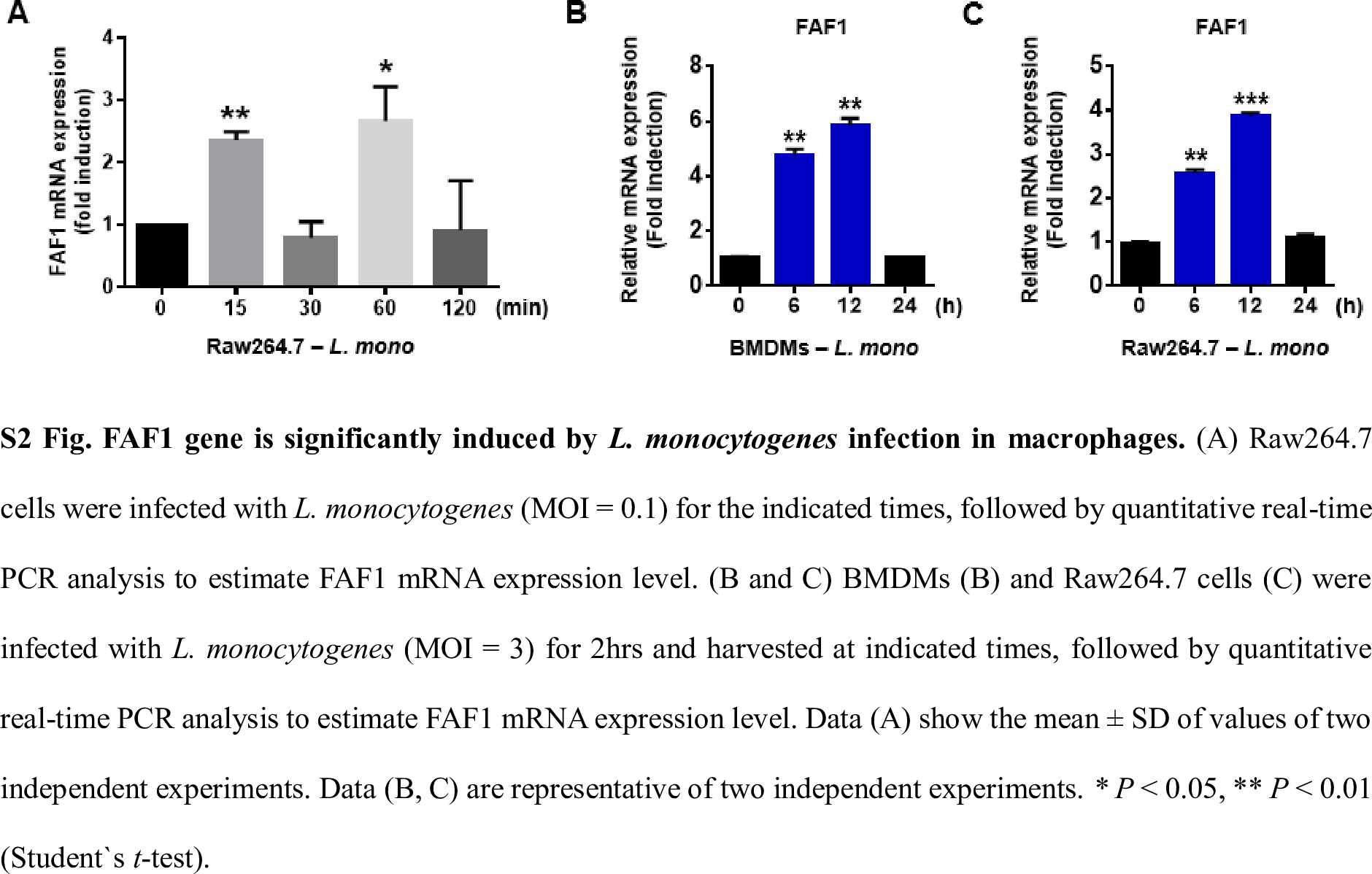

Supplement: S2 Fig — (A) Raw264.7 cells were infected with L. monocytogenes (MOI = 0.1) for the indicated times, followed by quantitative real-time PCR analysis to estimate FAF1 mRNA expression level. (B and C) BMDMs (B) and Raw264.7 cells (C) were infected with L. monocytogenes (MOI = 3) for 2hrs and harvested at indicated times, followed by quantitative real-time PCR analysis to estimate FAF1 mRNA expression level. Data (A) show the mean ± SD of values of two independent experiments. Data (B, C) are representative of two independent experiments. * P < 0.05, ** P < 0.01 (Student`s t-test). (TIF) [file ppat.1008004.s002.tif]

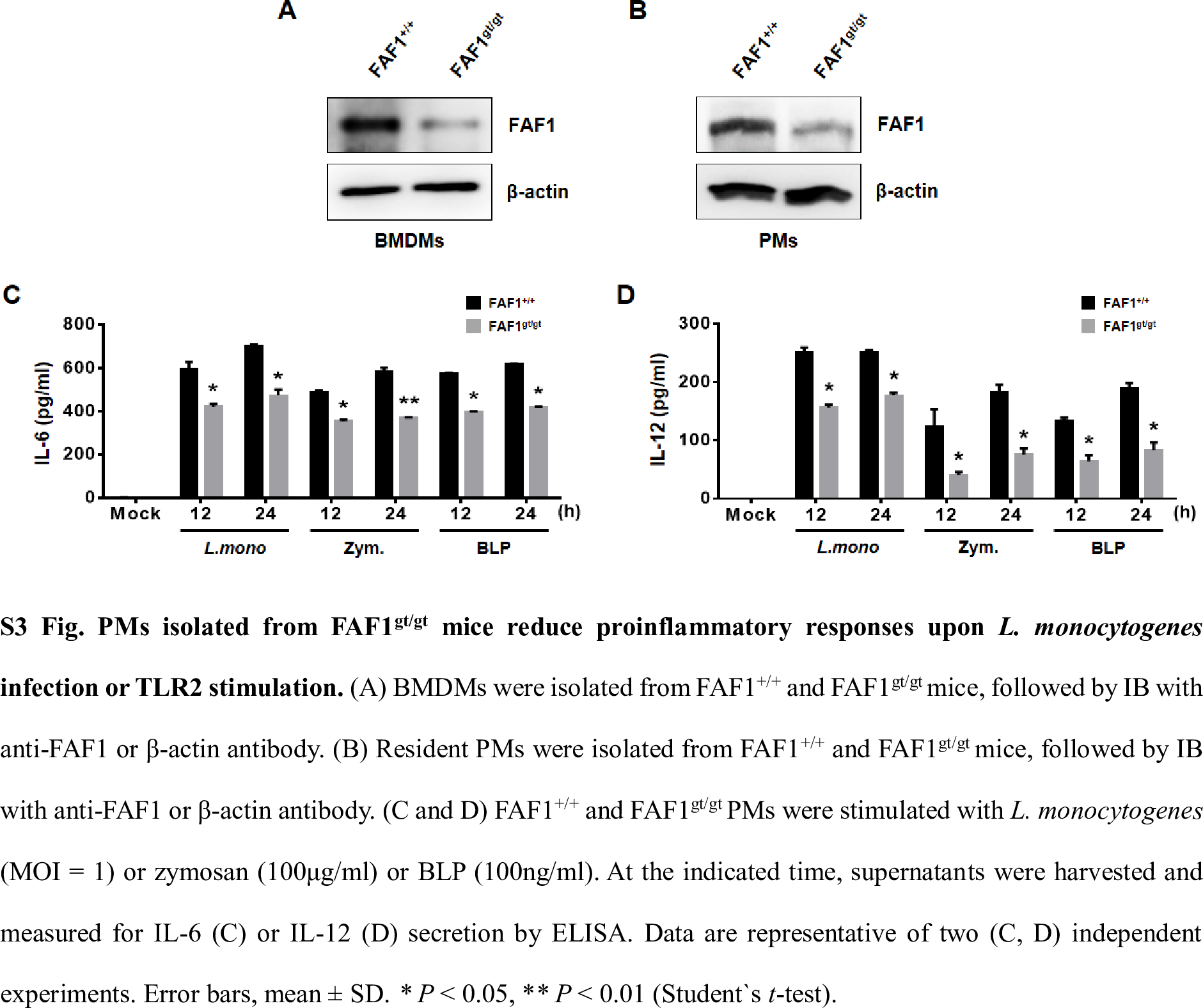

Supplement: S3 Fig — (A) BMDMs were isolated from FAF1+/+ and FAF1gt/gt mice, followed by IB with anti-FAF1 or β-actin antibody. (B) Resident PMs were isolated from FAF1+/+ and FAF1gt/gt mice, followed by IB with anti-FAF1 or β-actin antibody. (C and D) FAF1+/+ and FAF1gt/gt PMs were stimulated with L. monocytogenes (MOI = 1) or zymosan (100μg/ml) or BLP (100ng/ml). At the indicated time, supernatants were harvested and measured for IL-6 (C) or IL-12 (D) secretion by ELISA. Data are representative of two (C, D) independent experiments. Error bars, mean ± SD. * P < 0.05, ** P < 0.01 (Student`s t-test). (TIF) [file ppat.1008004.s003.tif]

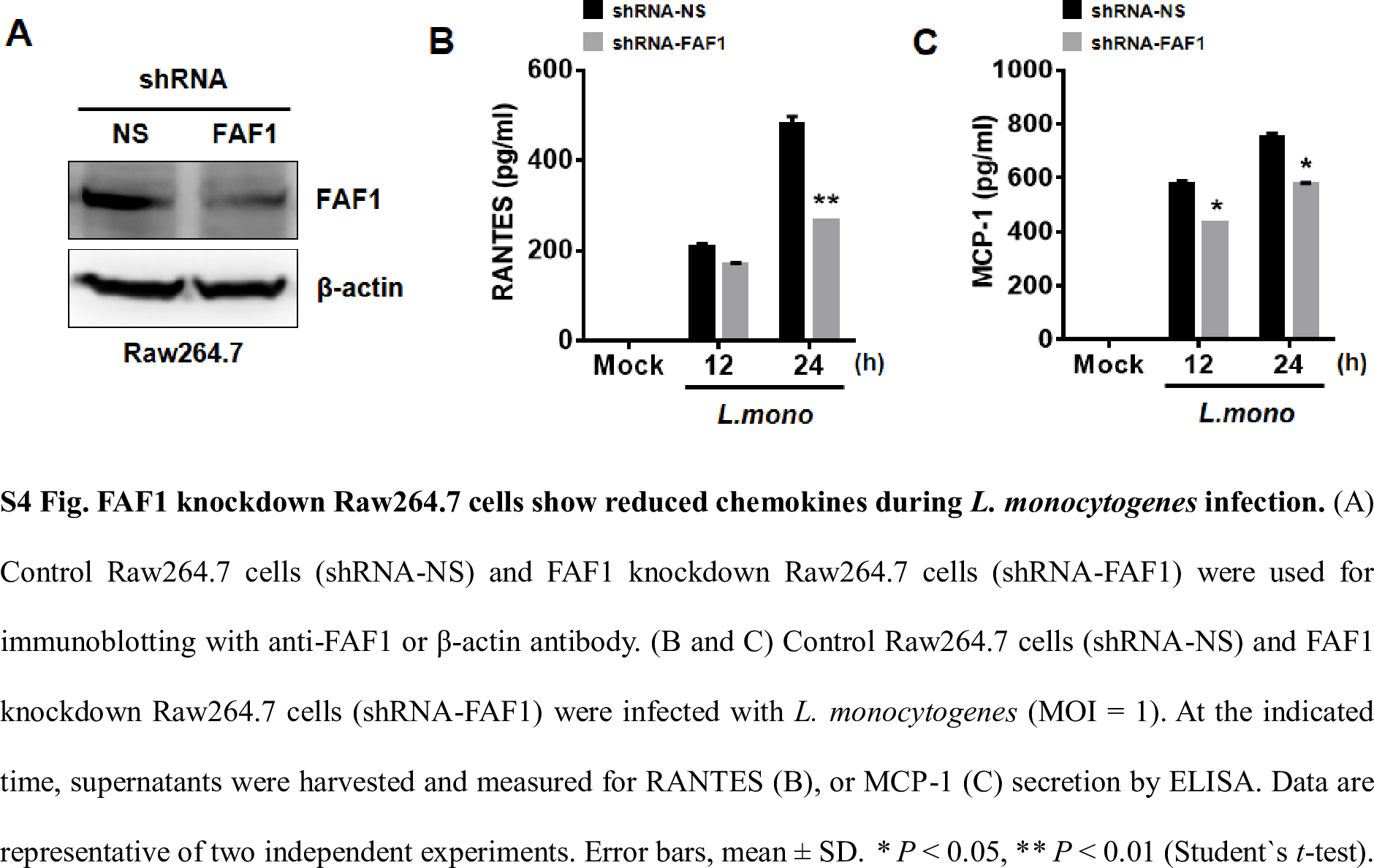

Supplement: S4 Fig — (A) Control Raw264.7 cells (shRNA-NS) and FAF1 knockdown Raw264.7 cells (shRNA-FAF1) were used for immunoblotting with anti-FAF1 or β-actin antibody. (B and C) Control Raw264.7 cells (shRNA-NS) and FAF1 knockdown Raw264.7 cells (shRNA-FAF1) were infected with L. monocytogenes (MOI = 1). At the indicated time, supernatants were harvested and measured for RANTES (B), or MCP-1 (C) secretion by ELISA. Data are representative of two independent experiments. Error bars, mean ± SD. * P < 0.05, ** P < 0.01 (Student`s t-test). (TIF) [file ppat.1008004.s004.tif]

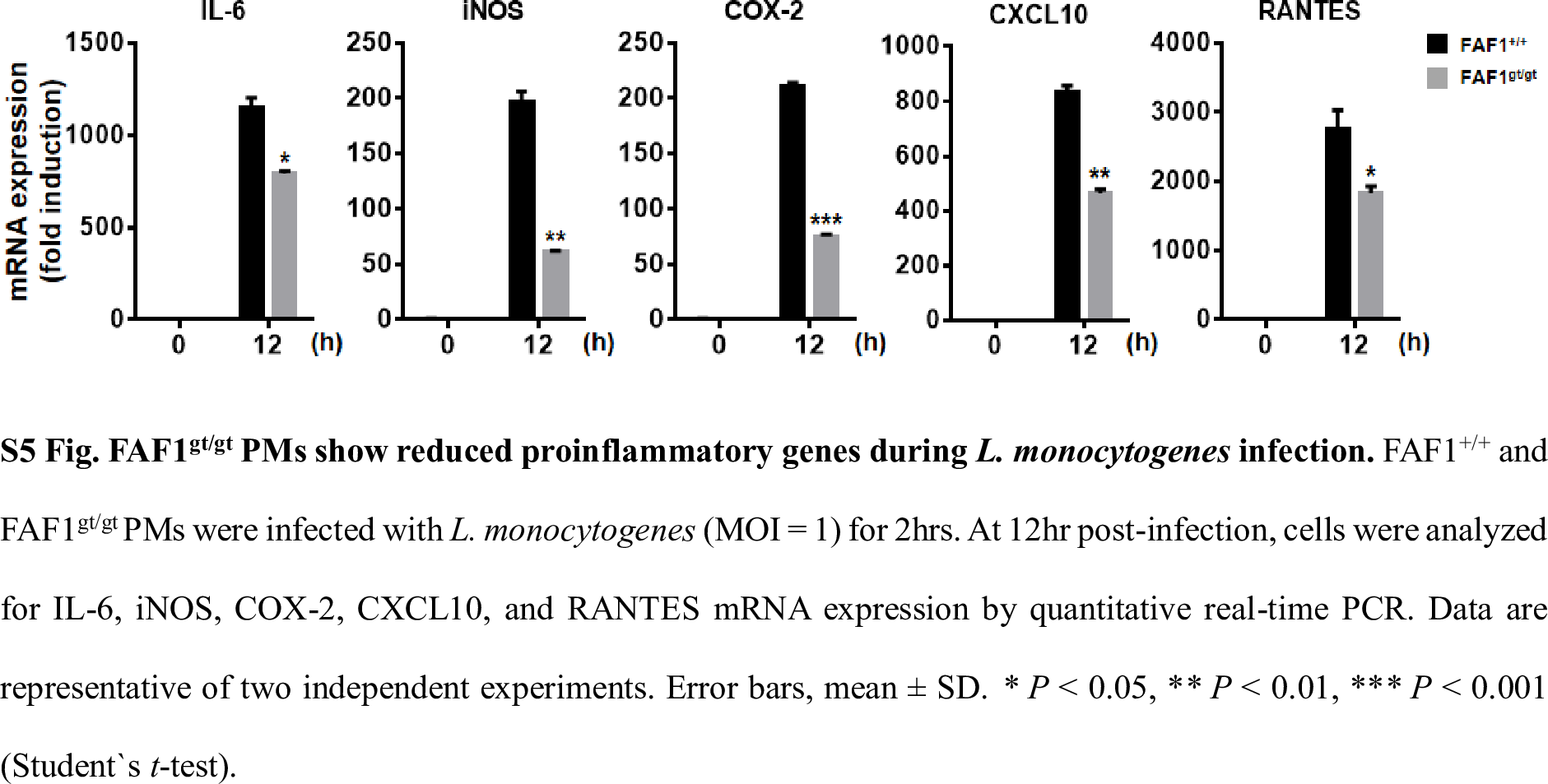

Supplement: S5 Fig — FAF1+/+ and FAF1gt/gt PMs were infected with L. monocytogenes (MOI = 1) for 2hrs. At 12hr post-infection, cells were analyzed for IL-6, iNOS, COX-2, CXCL10, and RANTES mRNA expression by quantitative real-time PCR. Data are representative of two independent experiments. Error bars, mean ± SD. * P < 0.05, ** P < 0.01, *** P < 0.001 (Student`s t-test). (TIF) [file ppat.1008004.s005.tif]

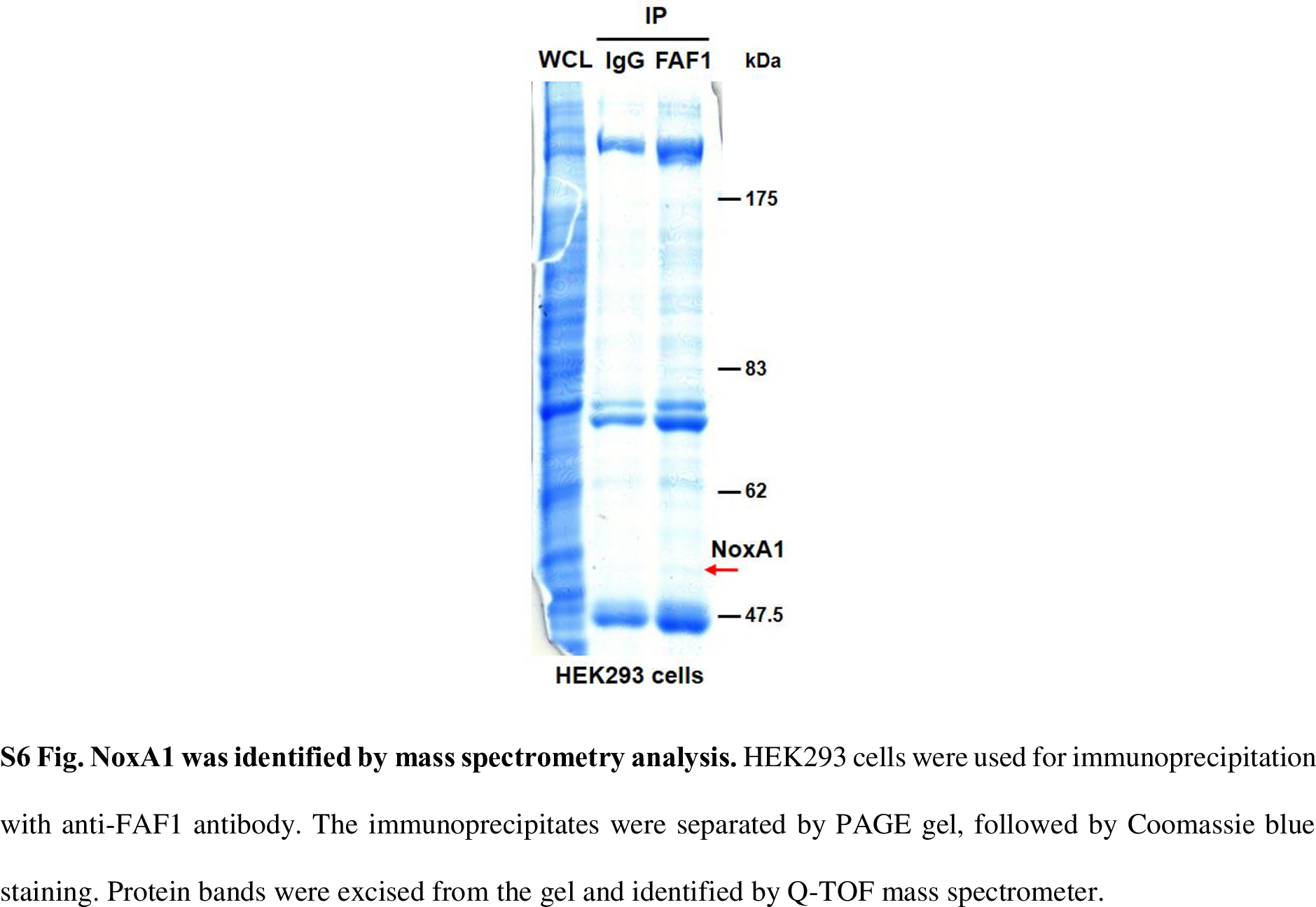

Supplement: S6 Fig — HEK293 cells were used for immunoprecipitation with anti-FAF1 antibody. The immunoprecipitates were separated by PAGE gel, followed by Coomassie blue staining. Protein bands were excised from the gel and identified by Q-TOF mass spectrometer. (TIF) [file ppat.1008004.s006.tif]

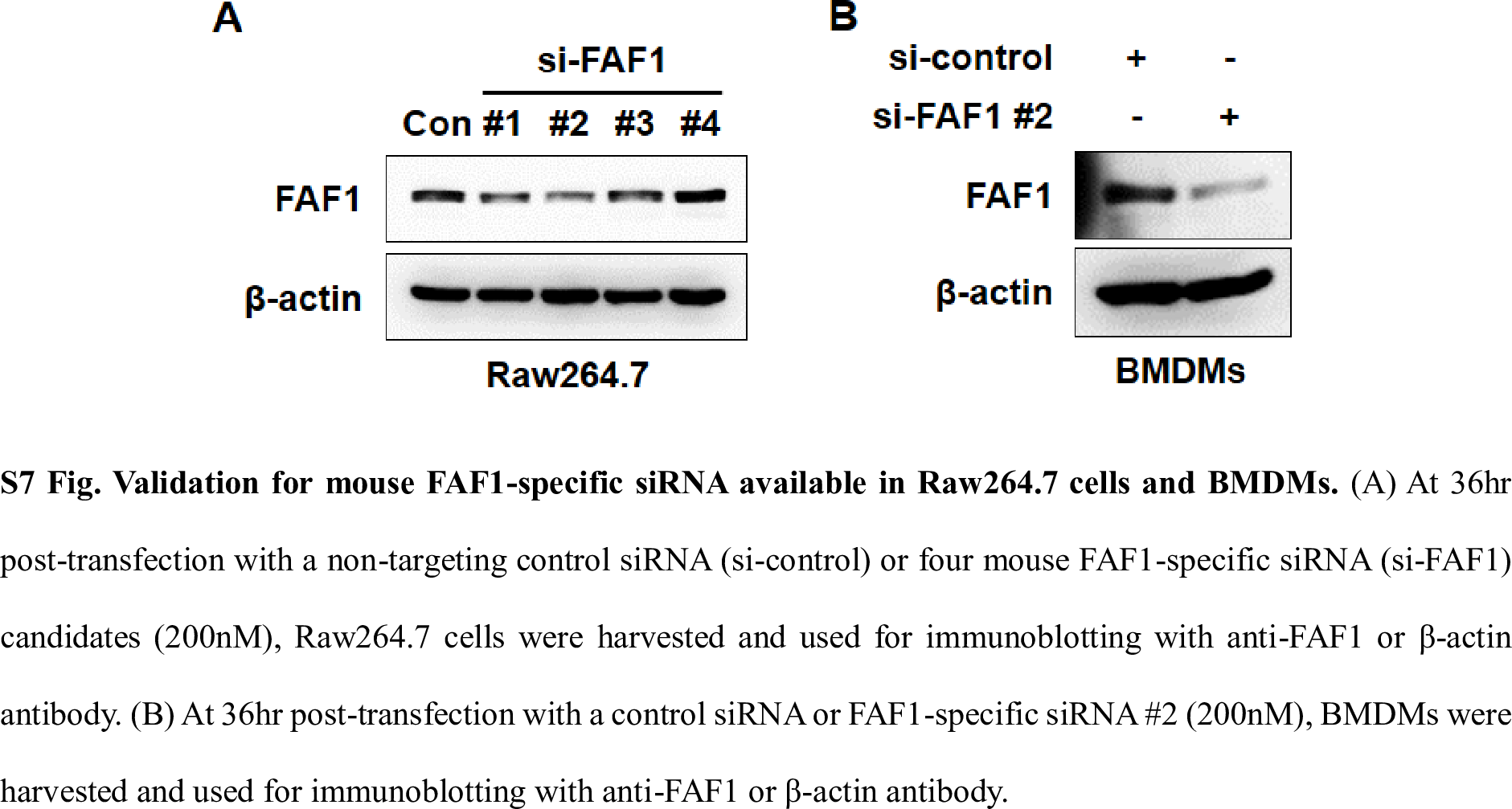

Supplement: S7 Fig — (A) At 36hr post-transfection with a non-targeting control siRNA (si-control) or four mouse FAF1-specific siRNA (si-FAF1) candidates (200nM), Raw264.7 cells were harvested and used for immunoblotting with anti-FAF1 or β-actin antibody. (B) At 36hr post-transfection with a control siRNA or FAF1-specific siRNA #2 (200nM), BMDMs were harvested and used for immunoblotting with anti-FAF1 or β-actin antibody. (TIF) [file ppat.1008004.s007.tif]

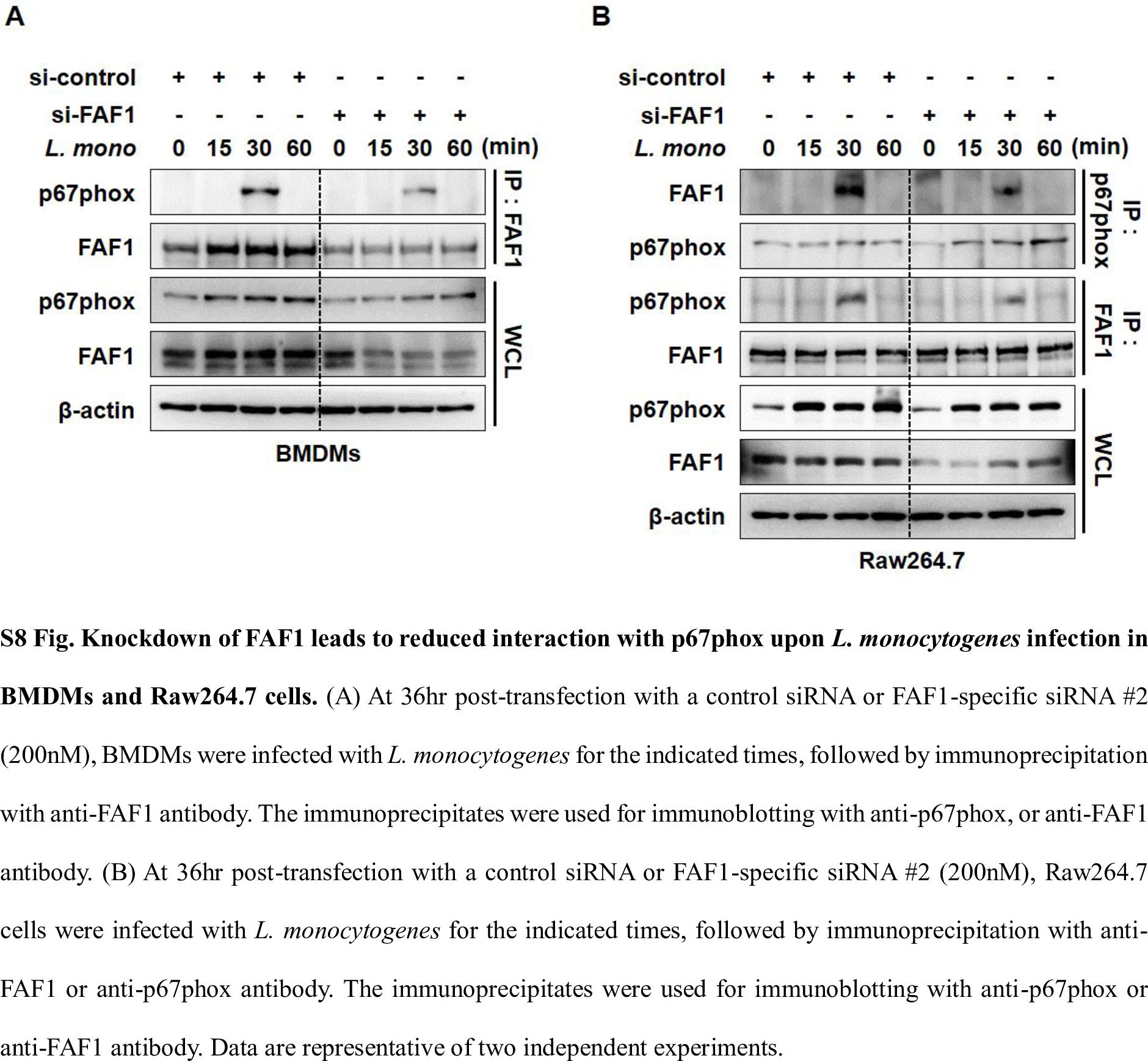

Supplement: S8 Fig — (A) At 36hr post-transfection with a control siRNA or FAF1-specific siRNA #2 (200nM), BMDMs were infected with L. monocytogenes for the indicated times, followed by immunoprecipitation with anti-FAF1 antibody. The immunoprecipitates were used for immunoblotting with anti-p67phox, or anti-FAF1 antibody. (B) At 36hr post-transfection with a control siRNA or FAF1-specific siRNA #2 (200nM), Raw264.7 cells were infected with L. monocytogenes for the indicated times, followed by immunoprecipitation with anti-FAF1 or anti-p67phox antibody. The immunoprecipitates were used for immunoblotting with anti-p67phox or anti-FAF1 antibody. Data are representative of two independent experiments. (TIF) [file ppat.1008004.s008.tif]

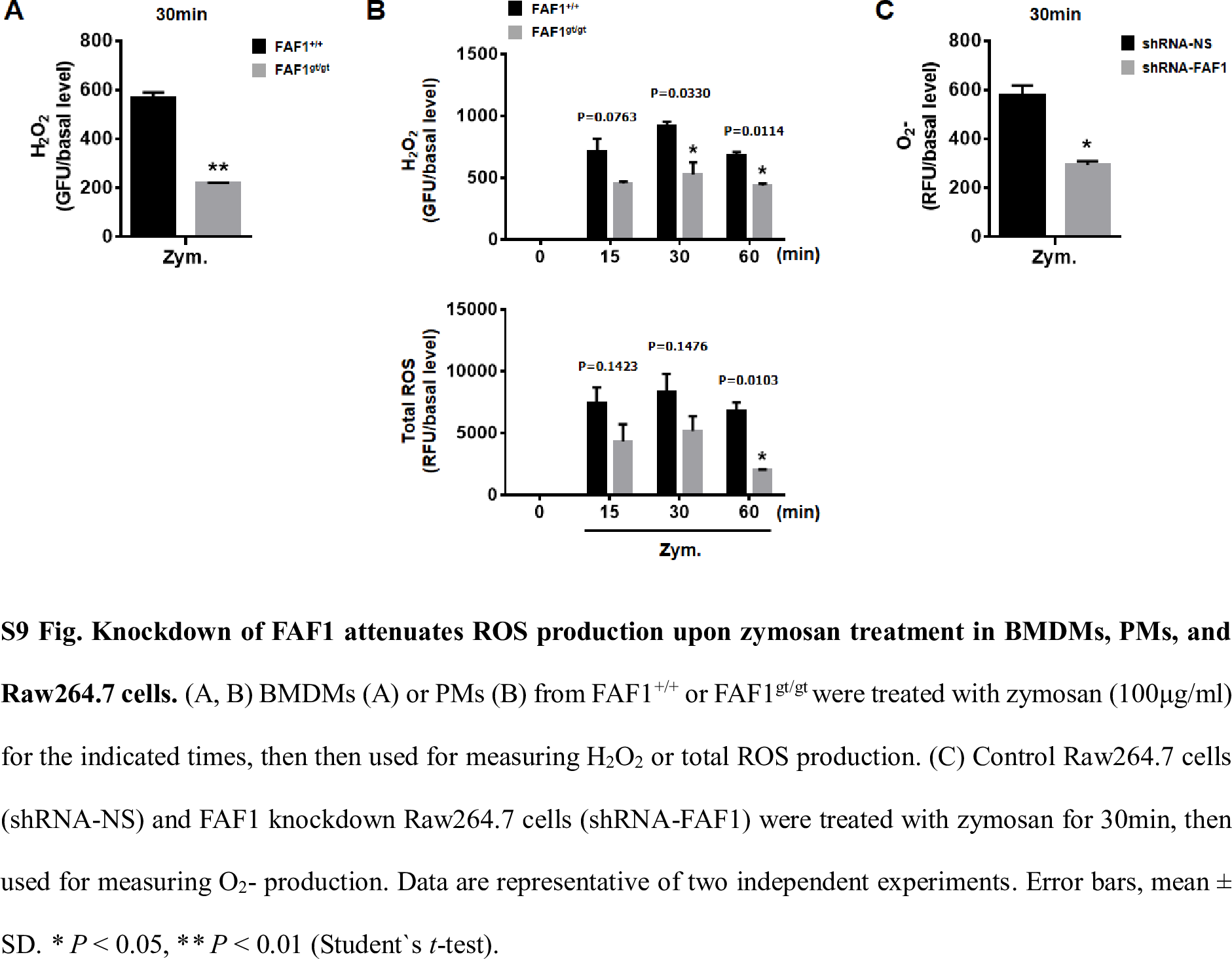

Supplement: S9 Fig — (A, B) BMDMs (A) or PMs (B) from FAF1+/+ or FAF1gt/gt were treated with zymosan (100μg/ml) for the indicated times, then then used for measuring H2O2 or total ROS production. (C) Control Raw264.7 cells (shRNA-NS) and FAF1 knockdown Raw264.7 cells (shRNA-FAF1) were treated with zymosan for 30min, then used for measuring O2- production. Data are representative of two independent experiments. Error bars, mean ± SD. * P < 0.05, ** P < 0.01 (Student`s t-test). (TIF) [file ppat.1008004.s009.tif]

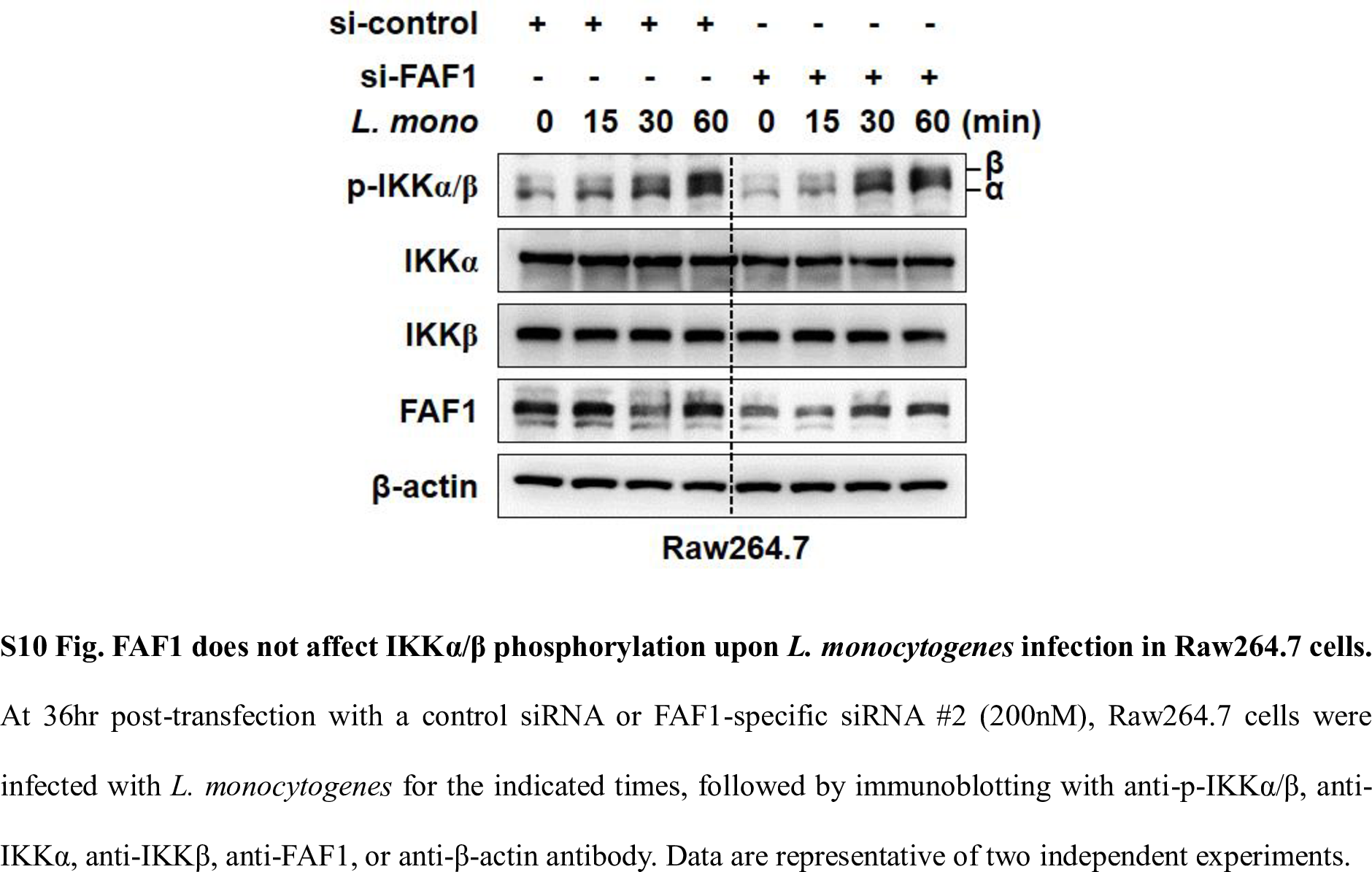

Supplement: S10 Fig — At 36hr post-transfection with a control siRNA or FAF1-specific siRNA #2 (200nM), Raw264.7 cells were infected with L. monocytogenes for the indicated times, followed by immunoblotting with anti-p-IKKα/β, anti-IKKα, anti-IKKβ, anti-FAF1, or anti-β-actin antibody. Data are representative of two independent experiments. (TIF) [file ppat.1008004.s010.tif]

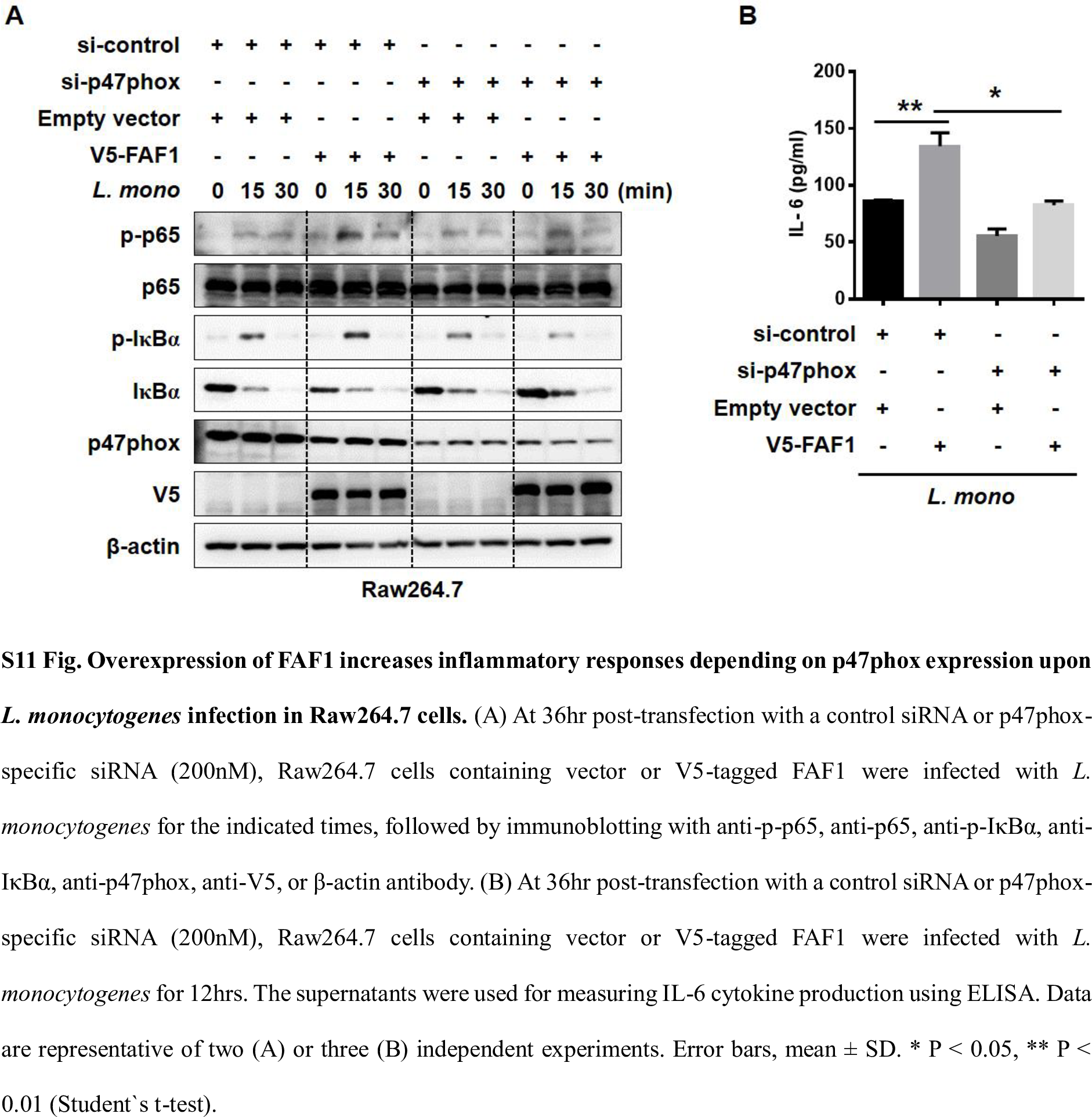

Supplement: S11 Fig — (A) At 36hr post-transfection with a control siRNA or p47phox-specific siRNA (200nM), Raw264.7 cells containing vector or V5-tagged FAF1 were infected with L. monocytogenes for the indicated times, followed by immunoblotting with anti-p-p65, anti-p65, anti-p-IκBα, anti-IκBα, anti-p47phox, anti-V5, or β-actin antibody. (B) At 36hr post-transfection with a control siRNA or p47phox-specific siRNA (200nM), Raw264.7 cells containing vector or V5-tagged FAF1 were infected with L. monocytogenes for 12hrs. The supernatants were used for measuring IL-6 cytokine production using ELISA. Data are representative of two (A) or three (B) independent experiments. Error bars, mean ± SD. * P < 0.05, ** P < 0.01 (Student`s t-test). (TIF) [file ppat.1008004.s011.tif]

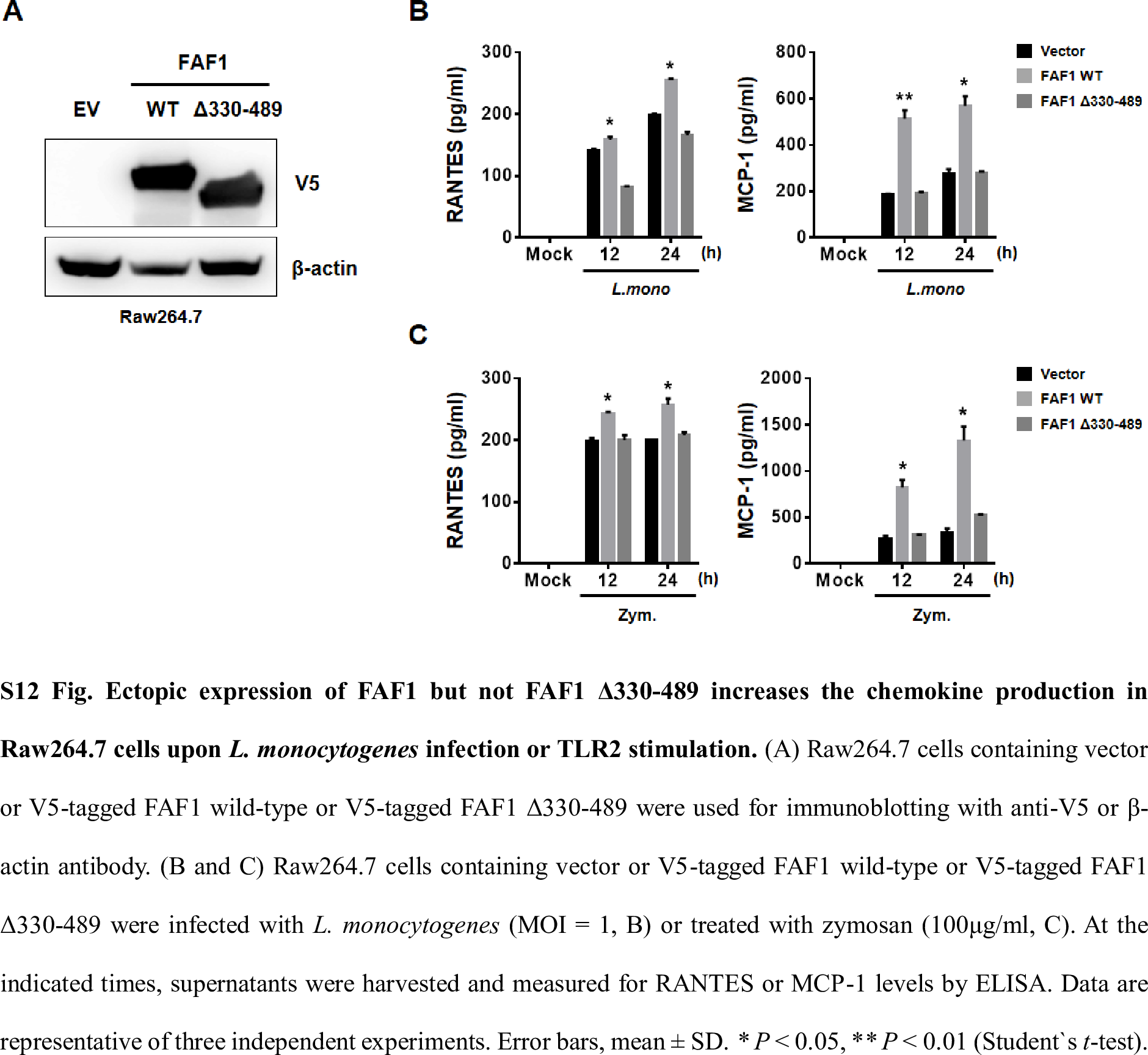

Supplement: S12 Fig — (A) Raw264.7 cells containing vector or V5-tagged FAF1 wild-type or V5-tagged FAF1 Δ330–489 were used for immunoblotting with anti-V5 or β-actin antibody. (B and C) Raw264.7 cells containing vector or V5-tagged FAF1 wild-type or V5-tagged FAF1 Δ330–489 were infected with L. monocytogenes (MOI = 1, B) or treated with zymosan (100μg/ml, C). At the indicated times, supernatants were harvested and measured for RANTES or MCP-1 levels by ELISA. Data are representative of three independent experiments. Error bars, mean ± SD. * P < 0.05, ** P < 0.01 (Student`s t-test). (TIF) [file ppat.1008004.s012.tif]

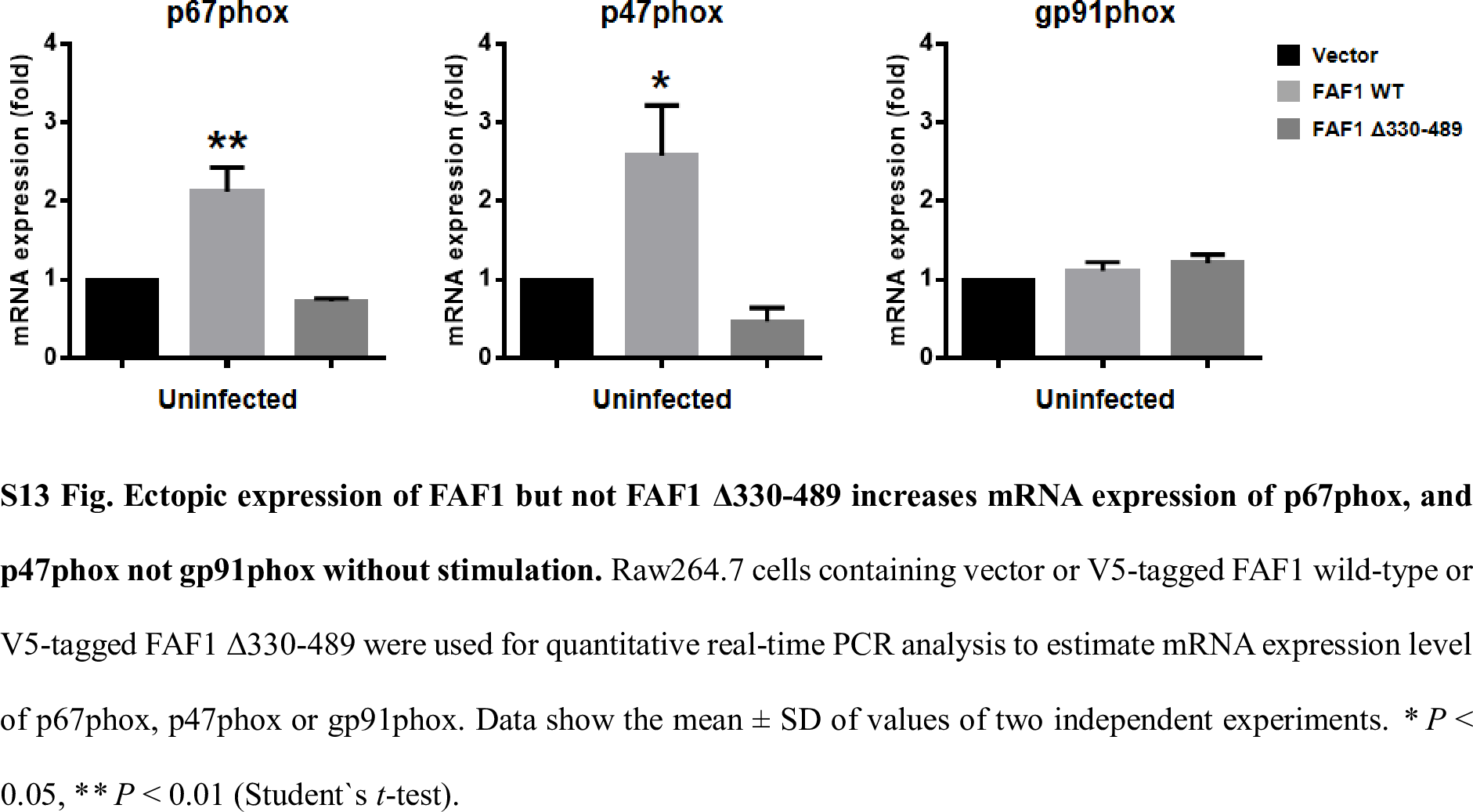

Supplement: S13 Fig — Raw264.7 cells containing vector or V5-tagged FAF1 wild-type or V5-tagged FAF1 Δ330–489 were used for quantitative real-time PCR analysis to estimate mRNA expression level of p67phox, p47phox or gp91phox. Data show the mean ± SD of values of two independent experiments. * P < 0.05, ** P < 0.01 (Student`s t-test). (TIF) [file ppat.1008004.s013.tif]

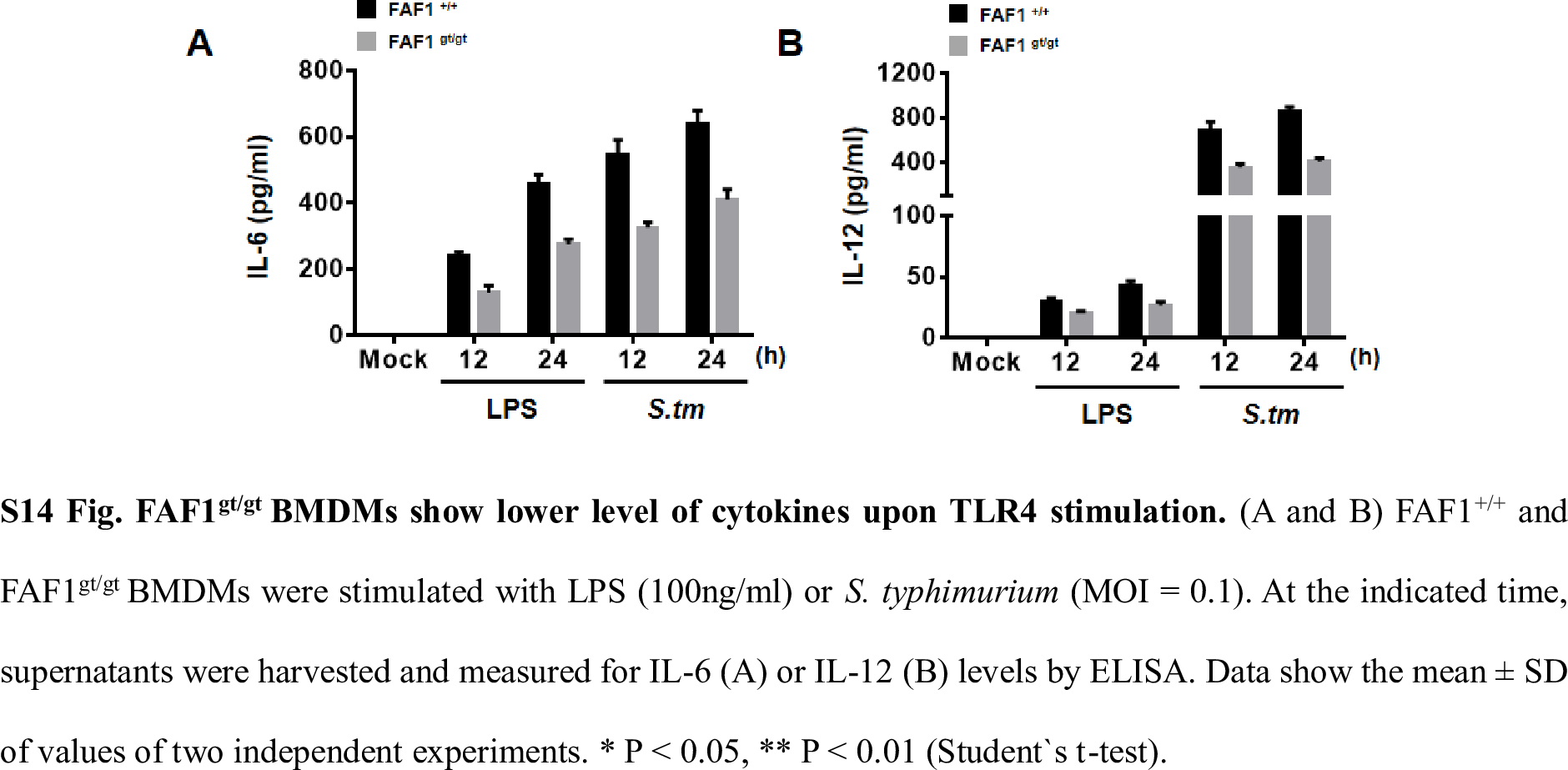

Supplement: S14 Fig — (A and B) FAF1+/+ and FAF1gt/gt BMDMs were stimulated with LPS (100ng/ml) or S. typhimurium (MOI = 0.1). At the indicated time, supernatants were harvested and measured for IL-6 (A) or IL-12 (B) levels by ELISA. Data show the mean ± SD of values of two independent experiments. * P < 0.05, ** P < 0.01 (Student`s t-test). (TIF) [file ppat.1008004.s014.tif]
